# Effectiveness of *N*‐Acetylcysteine for the Prevention of Contrast‐Induced Nephropathy: A Systematic Review and Meta‐Analysis of Randomized Controlled Trials

**DOI:** 10.1161/JAHA.116.003968

**Published:** 2016-09-23

**Authors:** Renfan Xu, Anyu Tao, Yang Bai, Youbin Deng, Guangzhi Chen

**Affiliations:** ^1^ Division of CardiologyDepartment of Internal MedicineTongji HospitalTongji Medical CollegeHuazhong University of Science and TechnologyWuhanChina; ^2^ Department of Medical UltrasoundTongji HospitalTongji Medical CollegeHuazhong University of Science and TechnologyWuhanChina

**Keywords:** contrast‐induced nephropathy, coronary angiography, meta‐analysis, *N*‐acetylcysteine, Nephrology and Kidney, Oxidant Stress, Percutaneous Coronary Intervention, Meta Analysis

## Abstract

**Background:**

Conflicting results have been obtained in trials that have evaluated the prophylactic efficacy of *N*‐acetylcysteine (NAC) pretreatment in the prevention of contrast‐induced nephropathy (CIN). In this meta‐analysis of randomized controlled trials, we aimed to assess the effectiveness of NAC treatment for the prevention of CIN.

**Methods and Results:**

PubMed, EMBASE, and the Cochrane Library were electronically searched from inception to January 2016 for all relevant studies. The weighted relative risk (RR) and corresponding 95% CI for incident CIN were estimated using random effects models. Standard methods for assessing statistical heterogeneity and publication bias were used. The study included 11 480 participants and 1653 cases of CIN. The incidence of CIN was 12.8% in the NAC group versus 16.0% in the control group (RR: 0.76, 95% CI: 0.66–0.88, *P*=0.0002). In the patients undergoing coronary angiography, the incidence of CIN in the NAC group versus the control group was 13.7% versus 17.2% (RR: 0.74, 95% CI: 0.63–0.87, *P*=0.0002); in those undergoing peripheral angiography, the incidence was 6.4% versus 5.8% (RR: 1.00, 95% CI: 0.42–2.40, *P*=1.00); in those undergoing computed tomography, the incidence was 7.7% versus 14.8% (RR: 0.51, 95% CI: 0.29–0.89, *P*=0.02).

**Conclusions:**

Our meta‐analysis showed an inverse and significant association between NAC supplementation and risk of CIN in patients undergoing coronary angiography and computed tomography, while a protective role for NAC in patients undergoing peripheral angiography was not obvious.

## Introduction

Contrast‐induced nephropathy (CIN) is a quite common and well‐known complication following the administration of iodinated contrast media and has become the third most common cause of hospital‐acquired acute kidney injury after hypotension and surgery.^1^ CIN is generally described as an increase in serum creatinine of 0.5 mg/dL or a 25% increase from the baseline value 48 hours after the procedure.^2^ CIN is reported to occur in as many as 14.5% of unselected patients undergoing coronary angiography/intervention,^1^ and the incidence may increase from 20% to 40% in high‐risk patients following the administration of a contrast agent.^3^ CIN is potentially preventable because the administration of radiocontrast agents is predictable and high‐risk populations have also been identified. Risk factors for CIN include preexisting renal dysfunction, diabetic nephropathy, congestive heart failure, reduced effective arterial volume, high‐dose administration of contrast agents, and concomitant administration of potentially nephrotoxic drugs, among others.^3^, ^4^ The development of CIN increases morbidity, mortality, and the cost of medical care, especially in patients requiring dialysis.^5^

The precise mechanism leading to CIN has not been fully elucidated. There is evidence that contrast agents reduce renal function through a combination of renal vasoconstriction with consequent hypoxia, and direct toxicity on tubular epithelial cells.^6^, ^7^ Reactive oxygen species associated with the administration of a contrast agent may play a vital role in the progression of CIN. Reactive oxygen species can act directly and indirectly in both the cortical and medullary microcirculation, resulting in vasoconstriction, antidiuresis, and antinatriuresis.^8^, ^9^ In addition, superoxide dismutase, a scavenger of reactive oxygen species, can inhibit the renal damage induced by contrast agents.^10^

*N*‐Acetylcysteine (NAC) is a thiol‐containing, cell‐membrane‐permeable antioxidant. The benefit of NAC supplementation for the prevention of CIN in patients with renal insufficiency undergoing contrast‐enhanced computed tomography (CT) was first reported by Tepel et al^11^ in 2000. In addition, Diaz‐Sandoval et al^12^ found that NAC has beneficial effects in patients undergoing cardiac catheterization. Further studies have also attempted to analyze the association between NAC administration and CIN risk in patients undergoing contrast agent injection; however, the results have not been consistent. Some studies have shown benefits similar to those of the previously mentioned reports in patients after NAC administration,^13^, ^14^ while other trials have returned conflicting results and raised doubts about the utility of NAC.^15^, ^16^

There are several possible mechanisms underlying the association between NAC administration and CIN risk. NAC has the potential to prevent CIN risk due to its potent antioxidant^17^ and vasodilating actions secondary to increased expression of nitric oxide synthase.^18^ On the cellular level, studies have shown that NAC administration inhibits renal cell apoptosis in a dose‐dependent manner, meaning that the larger the dose, the more is the benefit derived.^17^ In animal experiments, compared with the control group, NAC results in an increase in nitric oxide production, which has the effect of vasodilation and the attenuation of ischemic renal failure.^19^ In epidemiological studies, it was found that NAC could increase plasma levels of reduced glutathione, an oxygen free‐radical scavenger, and could inhibit oxidative stress in the postischemic kidney.^20^

There have been few approved therapies for CIN. The current standard of care involves only the use of intravenous hydration and low‐osmolality contrast media, but the benefit of this approach is limited.^21^ NAC is the most widely studied pharmacological therapy because of its low cost, ready availability, ease of administration by both the oral route and as an intravenous injection, potentially beneficial cardiac effects, and limited side effects. Thus far, there have been no definite results regarding efficacy of NAC in CIN prevention, and the results from the clinical trials and meta‐analysis were conflicting; consequently, definite suggestions for clinical physicians cannot be derived from these results. The current study presents a systematic review and meta‐analysis of randomized controlled clinical trials on the associations between NAC administration and CIN risk, mortality risk, and nephropathy requiring dialysis, and on changes in creatinine, the main clinical marker of renal dysfunction.

## Methods

### Search Strategy

Relevant studies were identified by searching PubMed, EMBASE, and Cochrane Library databases from their inception to January 2016 to identify the association between NAC supplementation and CIN risk using the following search terms: (*N*‐acetylcysteine or NAC or acetylcysteine) and (contrast media or contrast agent or contrast‐induced nephropathy or contrast‐associated nephropathy or radiocontrast nephropathy or contrast nephrotoxicity or acute kidney failure or acute kidney injury). We further restricted the search to studies on humans and those written in English. Additional studies not captured by our database search were retrieved through a manual search of references from originally identified reviews and research reports. This process was repeated until no additional articles were identified. Because this was an analysis of previously published data, this study did not undergo or require Institutional Review Board approval.

### Study Selection

To be included in the analysis, a trial had to fulfill the following criteria: (1) randomized controlled trials involving adult patients undergoing coronary angiography or peripheral angiography or CT that assessed the anti‐CIN efficacy of NAC supplementation; (2) use of NAC as monotherapy or only in combination with hydration, with a control group that received placebo or hydration; (3) definition of CIN as an absolute increase in serum creatinine of ≥0.5 mg/dL (44 mmol/L) or a relative increase of ≥25% from the baseline value after the administration of contrast media; and (4) studies published in English. Exclusion criteria were as follows: (1) patients treated with both NAC and other drugs (except hydration); (2) trials with abstracts only; and (3) patients undergoing renal replacement or those with coexisting cancer or malignant disease.

### Data Extraction and Quality Assessment

Two investigators (X.R.F. and C.G.Z.) independently reviewed all relevant articles and identified eligible studies. Disagreements or uncertainties were resolved by consensus. The following data were extracted from each study: first author's name, publication year, geographic region, sample size, subject characteristics (age, sex, and baseline renal function), definition of CIN, dosage of NAC and contrast agent, administration route (oral or intravenous), and the intervention in the control group. The primary outcome was the development of CIN, defined as an absolute increase in the serum creatinine concentration of at least 0.5 mg/dL or a >25% from the baseline value that occurred within 2 to 5 days after contrast injection. In case of trials in which the incidence was reported in terms of both relative (by 25%) and absolute increase in creatinine (by 0.5 mg/dL) separately, the data for the relative increase were given preference on the basis of advantages of this approach.^22^ In addition, in case of trials in which the incidence was reported at 48 hours or other time periods, the 48 hours incidence was given precedence for this is the most common time point defined in CIN studies.^23^ The secondary outcomes included the incidence of mortality and nephropathy requiring dialysis and net changes in creatinine. Furthermore, in case of trials in which the creatinine change was reported at 48 hours and other time periods, we extracted the data of 48 hours change for this is the most common time point used in CIN studies.

The quality of the studies was assessed through the methods used by Moher et al.^24^ The criteria used for quality assessment were randomization, generation of random numbers, allocation concealment, double‐blinding, and follow‐up. One point was given for each area, with a possible score between 0 and 5. Trials were considered to be high‐quality with scores ≥4 and low‐quality with scores ≤3.

### Statistical Analysis

Statistical analysis was performed using the Review Manager 5.0 (Cochrane Collaboration) and STATA software version 12.0 (Stata Corporation). For dichotomous outcomes, the results were expressed as risk ratios (RRs) with 95% CIs. For continuous outcomes, the results were expressed as weighted mean difference with SD. For trials that did not report SDs, SD values were obtained from 95% CI, *P* values, or *t* or F statistics according to standard formulas.^25^ Heterogeneity was assessed using Cochrane Q statistic and the inconsistency index (I^2^), where a *P* value <0.10 or I^2^>50% was considered to be significant.^26^ If heterogeneity existed among the studies, the random effects model (the Dersimonian and Laird method) was used to calculate the pooled odds ratio. Otherwise, a fixed effect model (the Mantel–Haenszel method) was used for outcomes without obvious heterogeneity.^27^ Sensitivity analyses were performed to assess the stability of the results by removal of 1 study each time to identify the impact of individual studies on the pooled effect size. A *P* value <0.05 was considered to be statistically significant in this trial, unless otherwise specified. Publication bias was assessed by using funnel plots, Begg's test, and Egger's test.^28^

In addition, to further detect and evaluate clinically significant heterogeneity, subgroup analyses and univariate meta–regression analyses were conducted to explore potential effect modification by prespecified factors: different procedure method, NAC dosage, NAC administration route, baseline renal function, contrast agent dosage, Jadad score, and CIN definition. A *P* value <0.05 was considered to be statistically significant in this trial unless otherwise specified.

## Results

### Study Selection and Characteristics

**Figure 1:**
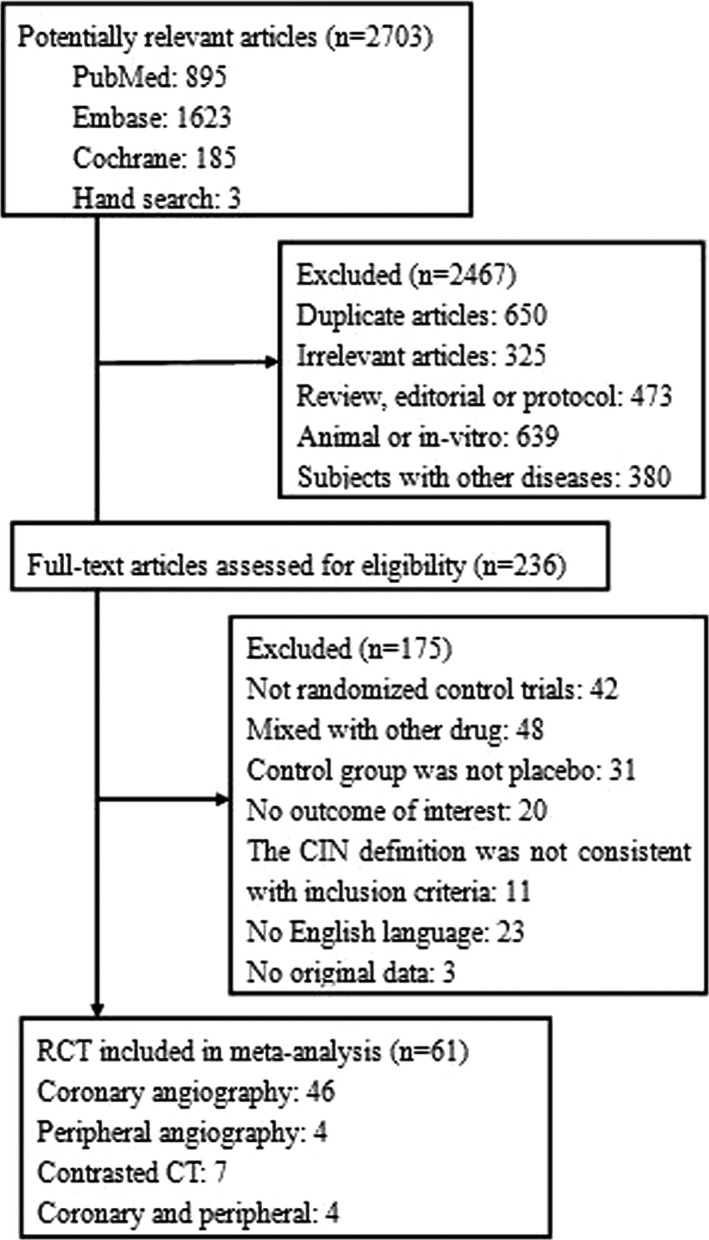
Study flow chart of meta‐analysis. CIN indicates contrast‐induced nephropathy; CT, computed tomography; RCT, randomized controlled trial.

We initially retrieved 2703 potentially relevant articles from the database, and 2467 articles were determined to be irrelevant after screening of the title or abstract. We conducted a detailed evaluation of the complete report for 236 trials proceeded to a detailed evaluation of the complete report, following which a further 175 articles were excluded. Finally, the 61 remaining articles were included in our meta‐analysis.^11^, ^12^, ^29^, ^30^, ^31^, ^32^, ^33^, ^34^, ^35^, ^36^, ^37^, ^38^, ^39^, ^40^, ^41^, ^42^, ^43^, ^44^, ^45^, ^46^, ^47^, ^48^, ^49^, ^50^, ^51^, ^52^, ^53^, ^54^, ^55^, ^56^, ^57^, ^58^, ^59^, ^60^, ^61^, ^62^, ^63^, ^64^, ^65^, ^66^, ^67^, ^68^, ^69^, ^70^, ^71^, ^72^, ^73^, ^74^, ^75^, ^76^, ^77^, ^78^, ^79^, ^80^, ^81^, ^82^, ^83^, ^84^, ^85^, ^86^, ^87^ A flowchart describing the article selection process for this meta‐analysis is shown in Figure 1.

The 61 articles with 66 comparisons were published between 1996 and 2016 and yielded a cumulative total of 11 480 patients, 5757 of whom were randomly assigned to the NAC group and 5723 to the control group (Table 1). All included studies had reported the incidence of CIN. Forty‐six studies with 48 comparisons^1^ were conducted in patients who underwent coronary angiography, 4 studies with 5 comparisons 64, 72, 74, 75 in patients who underwent peripheral angiography, 7 studies with 7 comparisons^11^, ^52^, ^60^, ^70^, ^81^, ^85^, ^87^ in patients who underwent CT, and 4 studies with 6 comparisons^36^, ^44^, ^45^, ^62^ in patients who underwent both coronary and peripheral angiography. The outcome of CIN was assessed by the change in the serum creatinine level. Thirty studies with 34 comparisons defined CIN as either >0.5 mg/dL or a 25% increase in the serum creatinine level,^2^ 13 studies with 13 comparisons^3^ defined CIN as >0.5 mg/dL increase in serum creatinine, and 18 studies with 19 comparisons^4^ defined CIN as a >25% increase in the serum creatinine level. The interventions used for all studies were NAC supplementation of varying dosages and treatment methods. The dosage of NAC supplements ranged from 600 to 16 000 mg. Twenty‐one studies with 23 comparisons^5^ chose to administer a total NAC dosage larger than 2400 mg, and 40 studies with 43 comparisons^6^ preferred a total supplementation dosage between 600 and 2400 IU. Forty‐six studies with 49 comparisons^7^ preferred oral supplementation strategies, 14 studies with 15 comparisons^8^ preferred intravenous route strategies, and the other 2 comparisons^32^, ^71^ selected both oral and intravenous strategies. Forty‐one studies with 44 comparisons^9^ enrolled patients with renal insufficiency at the baseline (serum creatinine ≥1.2 mg/dL), whereas the other 20 studies with 22 comparisons^10^ did not diagnose renal impairment. Twenty studies with 22 comparisons^11^ performed the injection of high‐dose contrast agent (>150 mL), while another 36 studies with 38 comparisons^12^ performed the injection of low‐dose contrast agent (<150 mL). Thirty studies with 33 comparisons^13^ were high‐quality trials, and 31 studies with 33 comparisons^14^ were low‐quality trials. Forty studies with 45 comparisons^15^ use isotonic saline as control group and 15 studies with 16 comparisons^16^ use hypotonic saline as control group. Four secondary outcome measures were examined: CIN risk in patients with diabetes mellitus (14 studies),^17^ creatinine (53 studies)^18^ nephropathy requiring dialysis (33 studies),^19^ and mortality (32 studies).^20^

**Table 1:** Baseline Characteristics of Studies Included in the Meta‐Analysis

First Author	Mean Baseline SCr (mg/dL)	Procedure Method	CIN Definition (SCr increase)	Contrast Volume (mL)	NAC dosage (mg)	NAC Route	Control Arm	Jadad Score
ACT^53^	1.2	C or P	≥25%	100	4800	PO	Placebo and 0.9% saline	5
Albabtain^29^	1.3	C	≥25% or 0.5 mg/dL	50	2400	PO	0.9% saline	3
Allaqaband^30^	2.1	C	≥0.5 mg/dL	124 (mean)	2400	PO	0.45% saline	3
Amini^31^	1.7	C	≥25% or 0.5 mg/dL	120	2400	PO	Placebo and 0.9% saline	5
Aslanger^32^	0.9	C	≥25%	199	9600	PO+ IV	Placebo and 0.9% saline	4
Azmus^33^	1.3	C	≥25% or 0.5 mg/dL	NA	3000	PO	Placebo and 0.9% saline	4
Baker^34^	1.8	C	≥25%	230	16000	IV	0.9% saline	3
Baskurt^35^	1.3	C	≥0.5 mg/dL	114	2400	PO	0.9% saline	3
Briguori^36^	1.5	C or P	≥25%	197	2400	PO	0.45% saline	2
Brueck^37^	1.5	C	≥0.5 mg/dL	110	1200	IV	Placebo and 0.9% saline	5
Carbonell (2007)^38^	0.9	C	≥25% or 0.5 mg/dL	187	2400	IV	Placebo and 0.45% saline	5
Carbonell (2010)^39^	1.9	C	≥25% or 0.5 mg/dL	159	2400	IV	Placebo and 0.9% saline	5
Castini^40^	1.5	C	≥25% or 0.5 mg/dL	203	2400	PO	0.9% saline	4
Coyle^41^	1.1	C	≥0.5 mg/dL	93	2400	PO	0.45% saline	3
Demir^85^	0.8	CT	≥25% or 0.5 mg/dL	100	1800	PO	0.9% saline	2
Diaz‐Sandoval^12^	1.5	C	≥25% or 0.5 mg/dL	185	2400	PO	Placebo and 0.45% saline	5
Droppa^42^	1.0	C	≥25%	191	7200	IV	Placebo and 0.9% saline	3
Durham^43^	2.3	C	≥0.5 mg/dL	81	2400	PO	Placebo and 0.45% saline	4
Erturk‐a^44^	1.5	C or P	≥25% or 0.5 mg/dL	125	7200	PO	0.9% saline	2
Erturk‐b^44^	1.5	C or P	≥25% or 0.5 mg/dL	125	7200	IV	0.9% saline	2
Ferrario^45^	1.6	C or P	≥25% or 0.5 mg/dL	174	2400	PO	Placebo and 0.9% saline	4
Fung^46^	2.3	C	≥25% or 0.5 mg/dL	128	2400	PO	0.9% saline	2
Goldenberg^47^	2.0	C	≥0.5 mg/dL	116	3600	PO	Placebo and 0.45% saline	5
Gomes^48^	1.3	C	≥0.5 mg/dL	102	2400	PO	Placebo and 0.9% saline	5
Gulel^49^	1.7	C	≥0.5 mg/dL	NA	2400	PO	0.9% saline	3
Gunebakmaz^50^	1.4	C	≥25% or 0.5 mg/dL	64	4800	PO	0.9% saline	2
Habib^51^	1.0	C	≥25% or 0.5 mg/dL	NA	4800	PO	Placebo and 0.9% saline	3
Hsu, 2007^86^	≥1.6	C	≥25% or 0.5 mg/dL	188	2400	PO	Placebo and 0.45% saline	4
Hsu et al, 2012^52^	1.3	CT	≥25% or 0.5 mg/dL	89	600	IV	0.9% saline	3
Jaffery^54^	1.1	C	≥25%	166	6000	IV	Placebo and 0.9% saline	4
Kay^55^	1.3	C	≥25%	125	2400	PO	Placebo and 0.9% saline	4
Kefer^56^	1.1	C	≥25% or 0.5 mg/dL	199	2400	IV	Placebo and 0.9% saline	4
Khalili^87^	1.4	CT	≥25%	140	2400	PO	0.9% saline	2
Kim^57^	1.0	C	≥25% or 0.5 mg/dL	209	2400	PO	0.9% saline	3
Kimmel^58^	1.6	C	≥25% or 0.5 mg/dL	203	2400	PO	Placebo and 0.45% saline	4
Kinbara^59^	1.0	C	≥0.5 mg/dL	144	2816	PO	0.9% saline	2
Kitzler^60^	1.4	CT	≥25%	100	2400	PO	Placebo and 0.45% saline	5
Koc^61^	1.4	C	≥25% or 0.5 mg/dL	130	2400	PO	0.9% saline	2
Kotlyar‐a^62^	2.3	C or P	≥25% or 0.5 mg/dL	87	600	IV	Placebo and 0.9% saline	5
Kotlyar‐b^62^	2.3	Cor P	≥25% or 0.5 mg/dL	88	1200	IV	Placebo and 0.9% saline	5
Kumar‐a^63^	1.0	C	≥25%	NA	2400	PO	0.9% saline	2
Kumar‐b^63^	1.1	C	≥25%	NA	2400	PO	0.9% saline	2
Lawlor‐a^64^	1.9	P	≥25% or 0.5 mg/dL	163	2400	PO	Placebo and 0.9% saline	4
Lawlor‐b^64^	1.9	P	≥25% or 0.5 mg/dL	160	2400	PO	Placebo and 0.9% saline	4
MacNeill^65^	1.9	C	≥25%	110	3000	PO	0.45% saline	3
Marenzi‐a^66^	1.0	C	≥25% or 0.5 mg/dL	264	3600	PO	Placebo and 0.9% saline	5
Marenzi‐b^66^	1.0	C	≥25% or 0.5 mg/dL	259	7200	PO	Placebo and 0.9% saline	5
Miner^67^	1.4	C	≥25%	347	4000 or 6000	PO	0.45% saline	3
Ochoa^68^	2.0	C	≥25% or 0.5 mg/dL	148	2000	PO	Placebo and 0.9% saline	4
Oldemeyer^69^	1.6	C	≥25% or 0.5 mg/dL	130	6000	PO	Placebo and 0.45% saline	5
Poletti^70^	1.7	CT	≥25%	125	1800	IV	Placebo and 0.45% saline	4
Prasad^71^	1.0	C	≥25% or 0.5 mg/dL	NA	4800	PO+ IV	Not receive NAC or placebo	3
Rashid^72^	1.3	P	≥25% or 0.5 mg/dL	143	2000	IV	Placebo and 0.9% saline	4
Reinecke^73^	1.4	C	≥0.5 mg/dL	190	2400	PO	5% glucose and 0.9% saline	2
Sadat^74^	1.1	P	≥25%	73	2400	PO	0.9% saline	2
Sandhu^75^	1.2	P	≥0.5 mg/dL	136	2400	PO	Placebo	3
Seyon^76^	1.5	C	≥25% or 0.5 mg/dL	140	2400	PO	Placebo and 0.9% saline	4
Shyu^77^	2.8	C	≥0.5 mg/dL	117	200 per kg	PO	Placebo and 0.45% saline	3
Tanaka^78^	0.8	C	≥25%	211	2820	PO	Placebo and Ringer's lactate solution	3
Tepel^11^	2.5	CT	≥0.5 mg/dL	75	2400	PO	Placebo and 0.45% saline	4
Thayssen^79^	0.9	C	≥25%	145	3600	PO	0.9% saline	3
Thiele^80^	0.9	C	≥25%	170	6000	PO	Placebo and 0.9% saline	4
Traub^81^	1.0	CT	≥25% or 0.5 mg/dL	114	3000	IV	Placebo and 0.9% saline	4
Webb^82^	1.6	C	≥25% or 0.5 mg/dL	120	500	IV	Placebo and 5% dextrose saline	4
Yang^83^	0.8	C	≥25% or 0.5 mg/dL	127	2400	PO	0.9% saline	3
Yeganehkhah^84^	1.1	C	≥25%	44	2400	PO	0.9% saline	3

C indicates coronary; CIN, contrast‐induced nephropathy; CT, contrast‐enhanced computed tomography; IV, intravenous; NA, not applicable; NAC, *N*‐acetylcysteine; P, peripheral; PO, orally; SCr, serum creatinine.

### Meta‐Analysis

**Figure 2:**
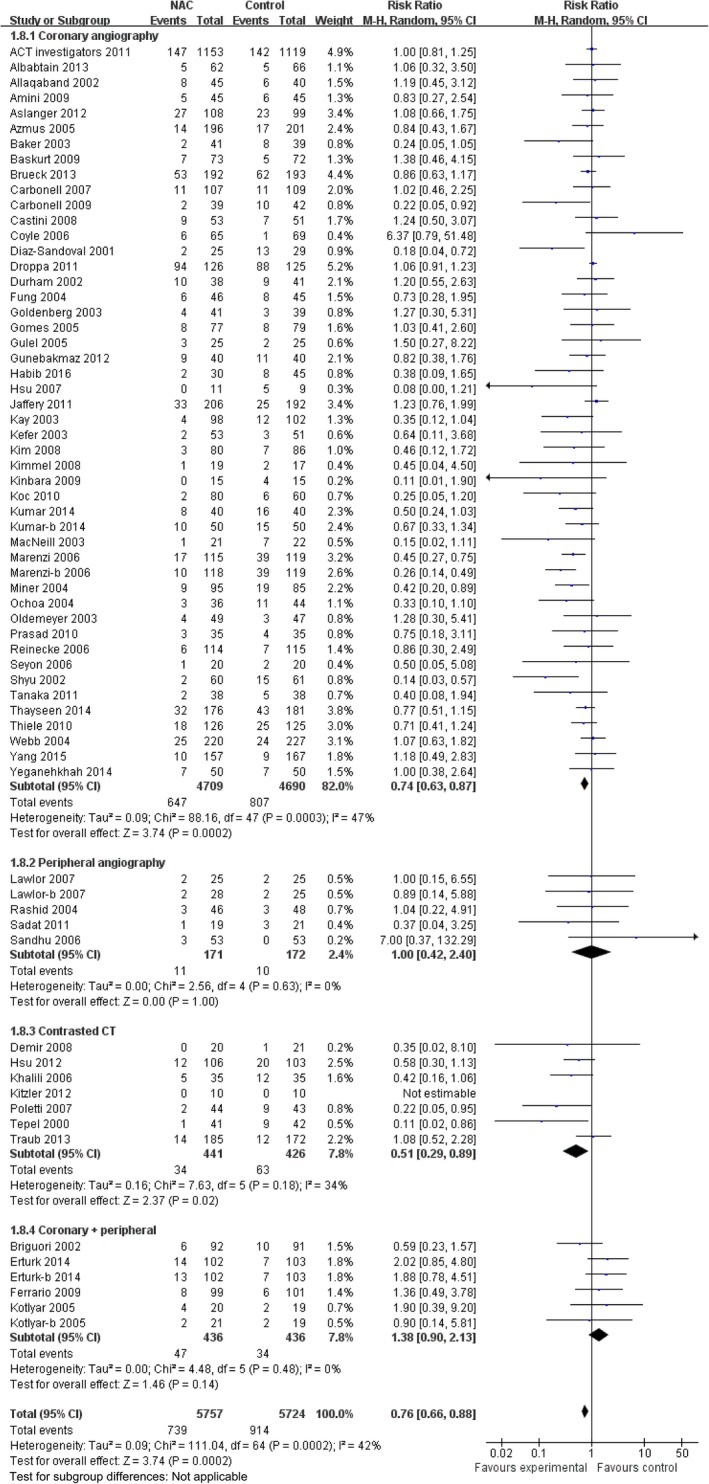
Forest plot of risk ratios and 95% CIs for the incidence of contrast‐induced nephropathy in patients assigned to NAC (*N*‐acetylcysteine) therapy vs control. CT indicates computed tomography.

In this meta‐analysis, there were 1653 CIN events among 11 480 included patients (14.4%). The incidence of CIN was 12.8% (739 of 5757) in the NAC group and 16.0% (914 of 5723) in the control group; in the pooled analysis using a random effects model, patients receiving NAC had a 24% lower risk of CIN than the control group (RR: 0.76, 95% CI: 0.66–0.88, *P*=0.0002), while the heterogeneity was significant (I^2^=42%; *P*=0.0002) (Figure 2). A sensitivity analysis was performed to confirm the robustness of our findings. We recalculated the pooled risk estimates for the remainder of the studies by omitting 1 study at a time, which resulted in little change in the observed risk estimates from 0.75 (95% CI 0.64–0.87) to 0.79 (95% CI 0.69–0.91).

### Subgroup Analysis

The following subgroups were tested for consistency of the major end points: NAC dosage, administration route, baseline renal function, contrast agent dosage, CIN definition, and Jadad score and control group. The results are shown in Table 2. In this subgroup analysis, an association between NAC intake and CIN risk was consistently observed in studies with different NAC dosage and Jadad score and control group, while the results were not consistent in other subgroups. NAC supplementation was more beneficial in patients with renal dysfunction, high doses of contrast agent, and oral administration of NAC. However, NAC intake had no effect in patients with normal renal function, low doses of contrast agent, and intravenous administration.

**Table 2:** Subgroup Analyses for the Effect of NAC Supplementation on CIN Risk

	No. of comparisons	No. (Case/Control)	Summary RR (95% CI)	*P‐*Value for Interaction	I^2^	*P‐*Value for Heterogeneity
All studies	66	5757/5723	0.76 (0.66, 0.88)	0.0002	42	0.0002
Procedure						
Coronary	48	4709/4690	0.74 (0.63, 0.87)	0.0002	47	0.0003
Peripheral	5	171/172	1.00 (0.42, 2.40)	1.00	0	0.63
CT	7	441/426	0.51 (0.29, 0.89)	0.02	34	0.18
Coronary+peripheral	6	436/436	1.38 (0.90, 2.13)	0.14	0	0.48
NAC dosage						
>2400 mg	23	3178/3124	0.76 (0.61, 0.95)	0.02	64	<0.0001
≤2400 mg	43	2579/2599	0.77 (0.65, 0.91)	0.003	11	0.27
Route						
Oral	49	4106/4104	0.69 (0.57, 0.83)	<0.0001	39	0.004
IV	15	1508/1485	0.94 (0.76, 1.15)	0.53	32	0.11
Oral+IV	2	143/134	1.04 (0.66, 1.64)	0.88	0	0.64
Renal function						
Dysfunction	44	3838/3795	0.77 (0.64, 0.93)	0.006	17	0.18
Normal	22	1919/1928	0.75 (0.59, 0.95)	0.02	56	0.0009
Contrast agent						
>150 mL	22	1718/1692	0.64 (0.48, 0.86)	0.03	40	<0.00001
≤150 mL	38	3663/3635	0.85 (0.71, 1.02)	0.08	29	0.05
Score						
>3	33	3468/3422	0.77 (0.63, 0.94)	0.01	43	0.005
≤3	33	2289/2301	0.75 (0.60, 0.93)	0.009	44	0.005
CIN definition						
25%+0.5	34	2390/2412	0.75 (0.60, 0.94)	0.01	33	0.04
25%	19	2528/2467	0.74 (0.60, 0.91)	0.004	53	0.005
0.5	13	839/844	0.92 (0.61, 1.39)	0.69	41	0.06
Control group						
Isotonic saline	45	4534/4501	0.75 (0.63, 0.90)	0.002	36	0.01
Hypotonic saline	16	763/754	0.48 (0.27, 0.86)	0.01	58	0.003

CIN indicates contrast‐induced nephropathy; CT, contrast‐enhanced computed tomography; IV, intravenous; NAC, *N*‐acetylcysteine; RR, risk ratio.

**Figure 3:**
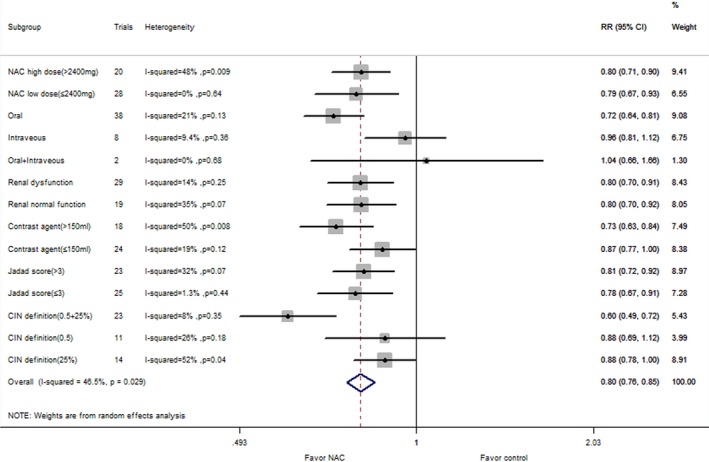
Subgroup analyses for the effect of NAC (*N*‐acetylcysteine) supplementation vs control on CIN (contrast‐induced nephropathy) risk for patients undergoing coronary angiography. RR, risk ratio.

Forty‐eight comparisons with 9399 patients reported a risk of CIN in coronary procedures. The corresponding relative risk was 13.7% in the NAC group versus 17.2% in the control group (RR: 0.74, 95% CI: 0.63–0.87, *P*=0.0002), with significant heterogeneity (*P*=0.0003, I^2^=47%; Figure 2). The subgroup analysis of NAC therapy on CIN risk for patients undergoing coronary angiography is listed in Figure 3. There were 5 comparisons with 343 reported rates of CIN risk in peripheral angiography. The incidence of CIN was 6.4% in the NAC group and 5.8% in the control group (RR: 1.00, 95% CI: 0.42–2.40; *P*=1.00). Low heterogeneity was seen with this analysis (I^2^=0%; *P*=0.63) (Figure 2). Seven comparisons with 867 patients reported an association between NAC intake and CIN risk in patients undergoing CT. The incidence of CIN was 7.7% in the NAC group versus 14.8% in the control group (RR: 0.51, 95% CI: 0.29–0.89, *P*=0.02).There was no evidence of heterogeneity (I^2^=34%; *P*=0.18) (Figure 2).

**Figure 4:**
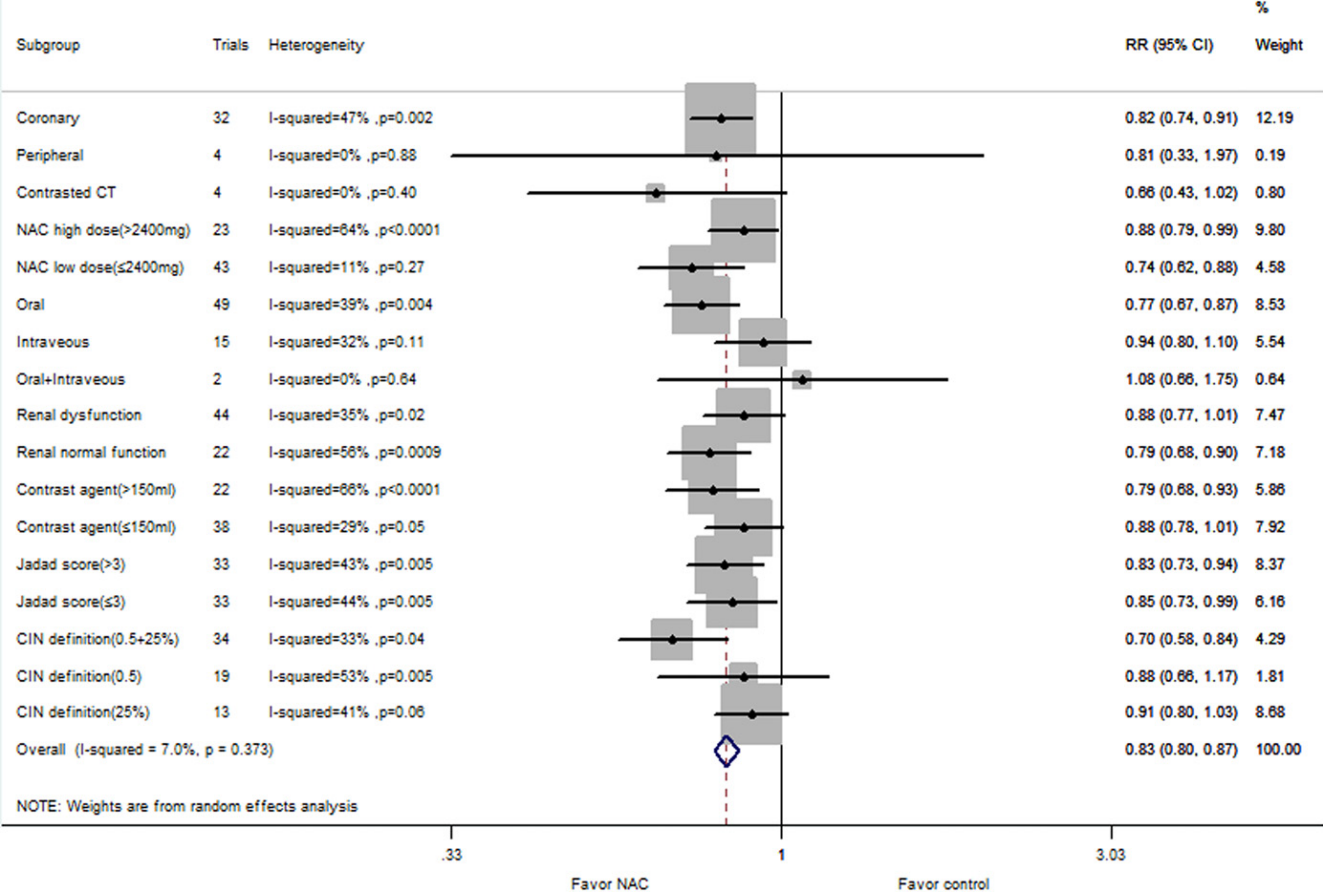
Subgroup analyses for the effect of NAC (*N*‐acetylcysteine) supplementation vs control (isotonic saline only) on CIN (contrast‐induced nephropathy) risk. CT indicates computed tomography; RR, risk ratio.

**Figure 5:**
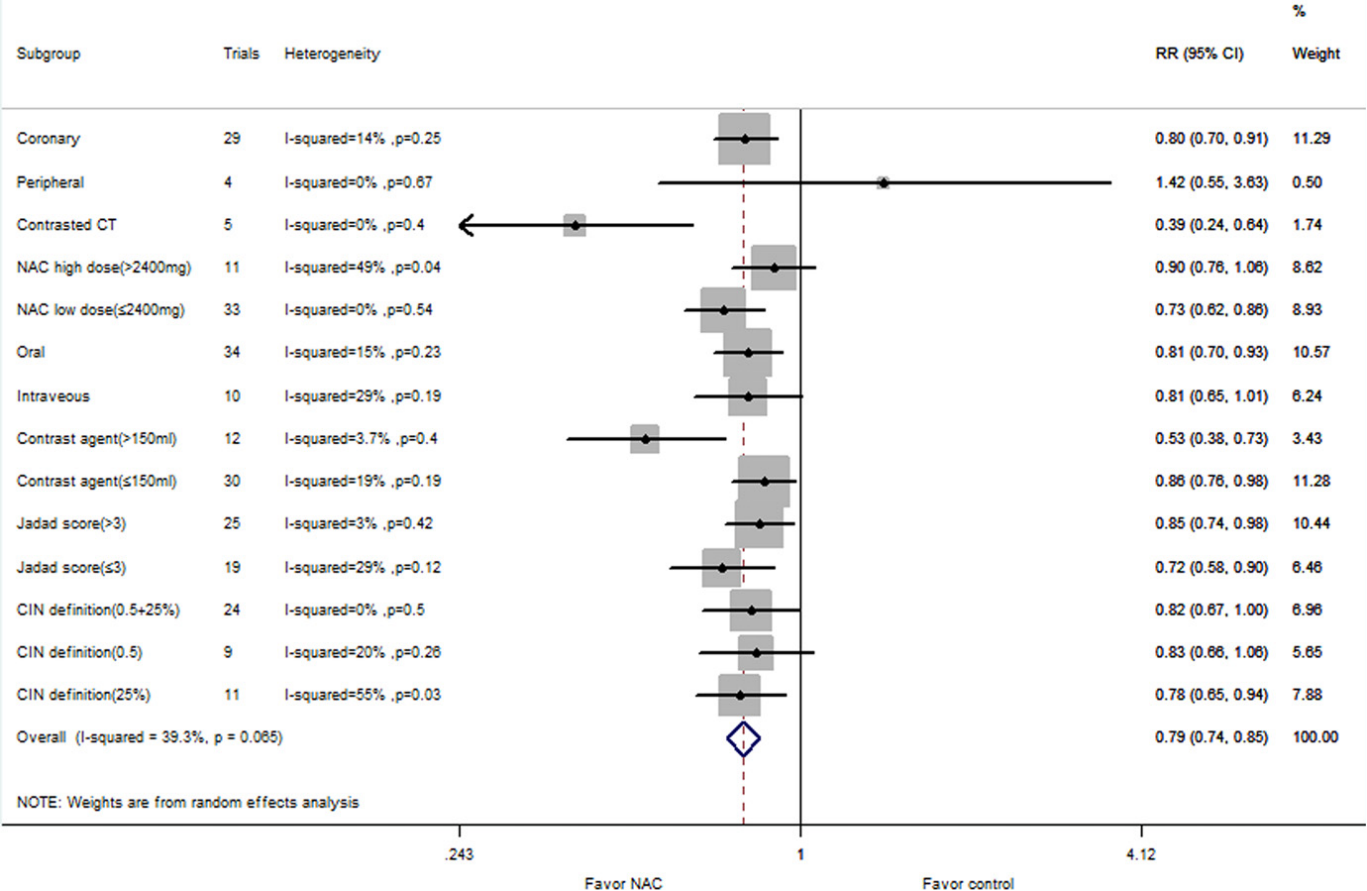
Subgroup analyses for the effect of NAC (*N*‐acetylcysteine) supplementation vs control on CIN (contrast‐induced nephropathy) risk in patients with renal dysfunction. CT indicates computed tomography; RR, risk ratio.

**Figure 6:**
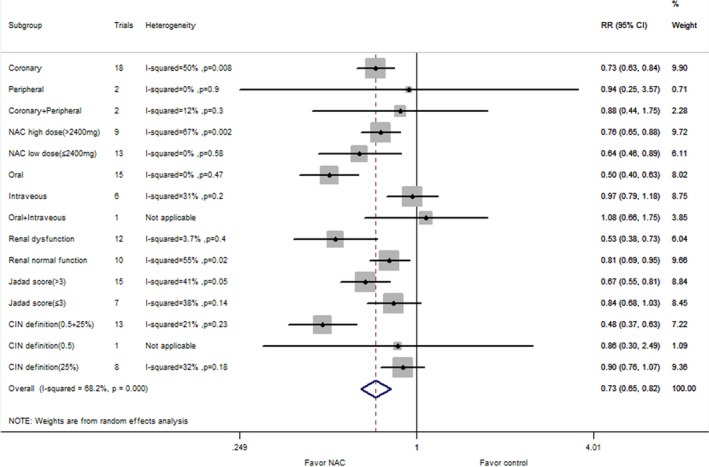
Subgroup analyses for the effect of NAC (*N*‐acetylcysteine) supplementation vs control on CIN (contrast‐induced nephropathy) risk in patients with high contrast agent. RR indicates risk ratio.

**Figure 7:**
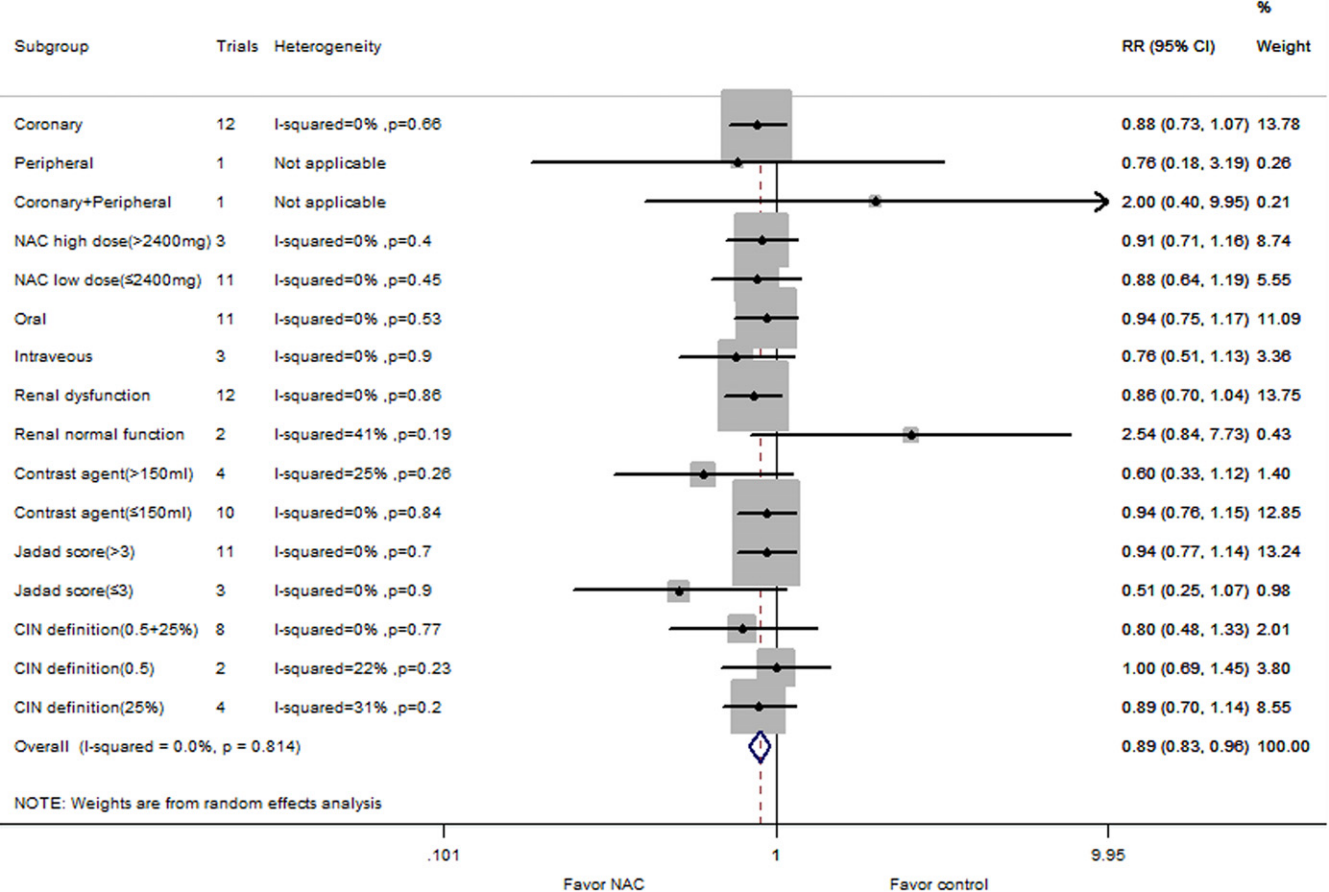
Subgroup analyses for the effect of NAC (*N*‐acetylcysteine) supplementation vs control on CIN (contrast‐induced nephropathy) risk in diabetes mellitus patients. RR indicates risk ratio.

A total of 45 comparisons used isotonic saline as control group. The corresponding relative risk was 13.9% in the NAC group versus 16.7% in the control group (RR: 0.75, 95% CI: 0.63–0.90, *P*=0.002), with significant heterogeneity (*P*=0.01, I^2^=36%; Figure 4). Sixteen comparisons used hypotonic saline as control group. The corresponding relative risk was 8.8% in the NAC group versus 16.2% in the control group (RR: 0.48, 95% CI: 0.27–0.86, *P*=0.01), with significant heterogeneity (*P*=0.003, I^2^=58%; Figure 4). Forty‐five comparisons with 7750 patients analyzed the CIN risk in patients with renal dysfunction (Figure 5). Compared with the control group, NAC administration significantly reduced risk of CIN (RR: 0.75, 95% CI: 0.63–0.89; *P*=0.001, I^2^=25%). Twenty comparisons with 3307 patients reported rates of CIN risk in patients with high doses of contrast agent (Figure 6). NAC significantly reduced the CIN risk compared with control (RR: 0.63, 95% CI: 0.46–0.86; *P*=0.003), with significant heterogeneity (I^2^=69%; *P*<0.00001). Fourteen comparisons with 2335 patients reported an association between NAC intake and CIN risk in patients with diabetes, although the results were not significant (RR: 0.91, 95% CI: 0.75–1.10, *P*=0.32). There was no evidence of heterogeneity (I^2^=0%; *P*=0.50) (Figure 7).

### Meta‐regression Analyses

**Figure 8:**
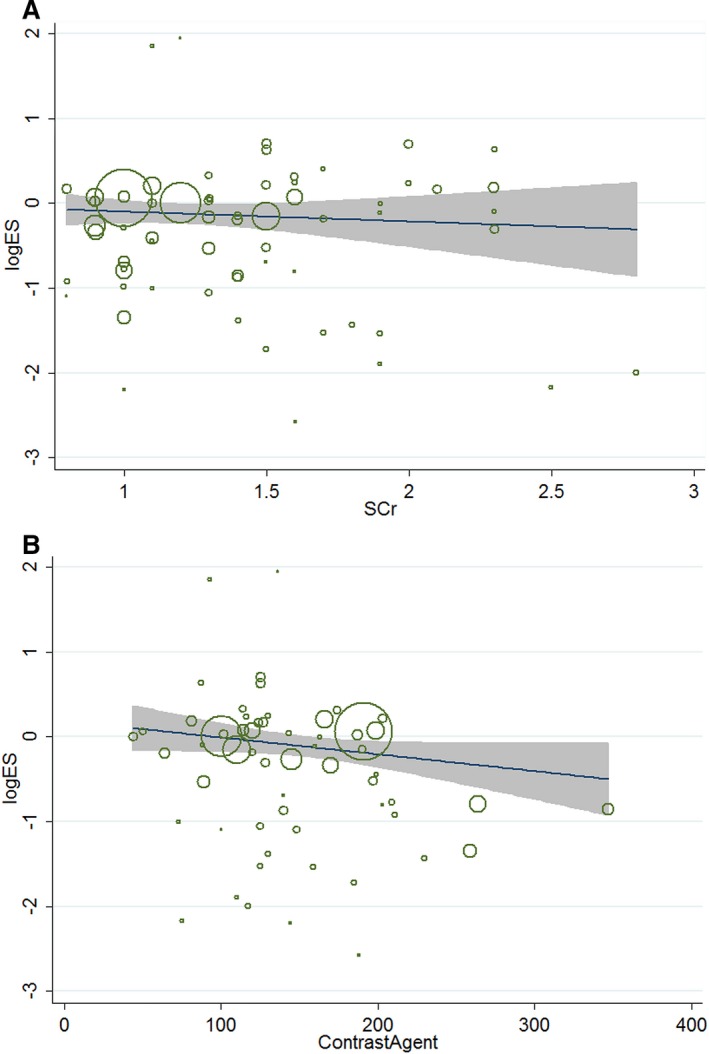
A, Relation between the risk of CIN and baseline levels of creatinine in 66 independent randomized controlled comparisons included in the meta‐analysis. Each circle represents a study, telescoped by its weight in the analysis. The relationship was not significant, suggesting that the impact of NAC on risk of CIN was consistent over the baseline levels of creatinine (*P*=0.855). B, Relation between the risk of CIN and contrast agent dosage in 60 independent randomized controlled comparisons included in the meta‐analysis. Each circle represents a study, telescoped by its weight in the analysis. The relationship was significant, suggesting that the impact of NAC on risk of CIN was consistent over the dosage of contrast agent (*P*=0.014). ES indicates effect size; SCr, serum creatinine.

The meta‐regression indicated that the impact of NAC on risk of CIN was consistent over baseline renal function (*P*=0.855) (Figure 8A). In contrast, meta‐regression by dosage of contrast agent did impact the relative reduction in risk of CIN for NAC versus control group (*P*=0.014) (Figure 8B), and this variate explained 32% of the heterogeneity across studies (residual I^2^=40.9%).

### Secondary Outcome

Compared with the control group, a significant reduction in blood creatinine level was observed in the NAC group (weighted mean difference: −0.08, 95% CI: −0.12 to −0.04, *P*<0.0001) with significant heterogeneity (I^2^=91%; *P*<0.00001) (Figure S1). The incidence of nephropathy requiring dialysis was extremely low, and only 43 cases were reported among 7168 randomized patients (0.44% in the NAC group, 0.76% in the placebo group). The overall results indicate that NAC admission does not significantly reduce the incidence of renal failure requiring renal dialysis (RR: 0.61, 95% CI: 0.32–1.17, I^2^=0%; Figure S2A). Mortality within 30 days occurred in 213 of the 6973 randomized patients, 2.8% in the NAC group versus 3.3% in the control group (RR: 0.85, 95% CI: 0.63–1.15, I^2^=13%) (Figure S2B).

### Study Quality and Publication Bias

The quality of these 61 randomized controlled trials was variable. Thirty studies with 33 comparisons were classified as high quality (Jadad score of 4 or 5), and the other 31 studies with 33 comparisons were classified as low quality (Jadad score of 2 or 3) (Table S1). The funnel plots of the studies were symmetric by visual in the current meta‐analysis (Figure S3). In addition, the Begg's test (*P*=0.08) and Egger's test (*P*=0.44) provided no evidence of publication bias.

## Discussion

This study is the most up‐to‐date comprehensive meta‐analysis to analyze the association between NAC intake and CIN risk in patients undergoing different interventions, including coronary angiography, CT, and peripheral angiography. In this review, NAC supplementation was shown to be associated with a significant decrease in CIN risk and blood creatinine level, both by overall analysis and across a number of stratified analyses based on key characteristics of study methods. However, NAC intake was not associated with reduction in mortality or nephropathy requiring dialysis. In addition, NAC supplementation could not reduce the CIN risk in patients with diabetes.

Observational prospective cohort studies and case–control studies have been performed to determine the protective role of NAC in the development of CIN, although the results have been conflicting. The protective effect of NAC intake on CIN risk was first pointed out by Tepel et al on patients undergoing contrasted CT,^11^ and this result was confirmed in studies on coronary angiography.^12^ However, a large observational nonrandomized prospective study involving 90 578 coronary angiography patients from the United States revealed that the use of NAC had no protective effect on CIN risk.^15^ In addition, some randomized controlled clinical trials have also demonstrated that NAC supplementation was not associated with CIN risk.^34^, ^36^, ^55^ Nonetheless, these results need to be interpreted with caution because the number of patients enrolled in most trials was too limited, at less than 200 patients; thus, the occurrence of CIN was limited and cannot represent the real epidemiological level of CIN. Our results were also consistent with the finding of a large meta‐analysis by Subramaniam et al.^88^, ^89^

Recent mechanistic studies have examined the effects of NAC on CIN risk after contrast agent injection and provided further evidence for the biological plausibility of these findings. The precise physiological insult underlying CIN may well involve the interplay of several pathogenic factors. First, contrast agent stimulates renal vasoconstriction and hyperviscosity, which cause hemodynamic changes in renal blood flow and hypoxia of the renal medulla.^6^ Second, contrast agent stimulates high oxidative stress in the renal medulla, which can reduce the level of nitric oxide (NO), an important regulator of medullary renal blood flow.^8^ Third, contrast agent has direct toxicity on renal cells.^6^ The efficacy of NAC on the inhibition of CIN risk was further supported by both in vivo and in vitro experiments. Through in vitro experiments, NAC supplementation was found to protect dose‐dependently cultured tubular cells that underwent short‐term incubation with very high concentrations (200 mg iodine/mL) of low‐ and iso‐osmolar contrast agent.^17^ From animal experiments, there is evidence that NAC pretreatment improves renal blood flow by direct renal vasodilation and by the release of renal prostaglandin E2 and renal cortical NO, which improve renal medullary blood flow.^90^ In clinical trials, in patients undergoing coronary angiography, NAC pretreatment did reduce the decline in urinary NO end products but did not affect lipid peroxidation, evaluated by urinary isoprostane.^20^

In this meta‐analysis, subgroup analysis was performed on the basis of our predefined variables to identify sources of heterogeneity. Baseline renal dysfunction and high doses of contrast agent were considered to be 2 important risk factors for CIN. NAC supplementation has a much more important benefit in CIN inhibition in patients with renal dysfunction and high contrast agent dosage than in patients with normal renal function and low dose of contrast agent. Our findings also suggest that subjects administered NAC though the oral route would experience increased benefits of CIN protection. It is important to note that intravenous NAC intake has an insufficient effect on the inhibition of CIN risk, although the tendency was obvious. Intravenous NAC might be more effective in administration, given its rapid onset of effect, higher peak serum NAC levels, and complete bioavailability; thus, more trials will be needed to analyze the exact mechanism by which NAC acts. We also adjusted for the type of fluid used in control group (isotonic versus hypotonic) and found that NAC intake has a sufficient effect on inhibition of CIN risk on both groups. Furthermore, a higher volume of contrast agent is more frequently needed if endoluminal therapy is required, and this is also a risk factor for CIN. In addition, subjects undergoing coronary angiography and CT, instead of peripheral angiography, may experience the maximum benefit of NAC on CIN inhibition. It is worth considering potential difference related to CIN resulting from different procedures. Patients undergoing coronary angiography are likely to have some baseline diseases, such as coronary disease, diabetes, or renal dysfunction, which are also CIN risk factors. However, only limited studies have analyzed the effect of NAC intake on CIN risk in patients undergoing peripheral angiography (5 studies) or CT (7 studies); therefore, more randomized controlled clinical trials will be required to make a definite conclusion.

The analysis of our secondary outcomes revealed a significant improvement in the blood creatinine level with NAC supplementation, which was consistent with our findings that CIN risk was significantly decreased. However, this analysis did not support the use of NAC to reduce the incidence of CIN in patients with diabetes, or nephropathy requiring dialysis. Diabetes mellitus has been regarded as an important risk factor for CIN. However, as only 2335 patients with diabetes, with 351 cases of CIN, were enrolled in this meta‐analysis, we could only observe a tendency instead of an obvious inhibition of CIN risk. In addition, NAC intake demonstrates only a tendency instead of significant protection from mortality.

This meta‐analysis has several significant strengths. First, to our knowledge, this study represents the largest available pooled analysis to date evaluating NAC efficacy for CIN prevention. The populations studied varied widely and covered several major risk factors for CIN, which enabled us to draw clinically relevant conclusions from different subsets of populations. Second, the trials included in this study were all randomized controlled trials, with careful monitoring and adjudication by blinded clinical events committees, which ensured the relatively high quality and the accurate information of the included studies. Finally, since the definition of CIN varied across studies, we chose change in the blood creatinine level rather than acute renal failure or requirement for dialysis as our primary outcome; thus, the differential misclassification of CIN attributable to recall bias was minimized.

Our analyses did have limitations. First, the sample sizes in most of these trials were relatively small, with numbers of patients less than 200; thus, meta‐analysis may have been underpowered to detect true differences. Second, a significant amount of unexplainable heterogeneity was detected in both primary and subgroup analyses, although our random effects model did account for this heterogeneity. It is possible that the baseline characteristics of the participants all contribute to variation in trial effects. Although the variables of contrast agent dosage account for part of the statistic heterogeneity, the residual heterogeneity remained at 40%. Third, we did not have access to patient‐level data to determine whether preexisting decreased renal function and other risk factors (eg, diabetes mellitus and advanced age) could influence the effect of NAC intake on CIN risk. Fourth, the follow‐up period for most included studies was only 48 or 72 hours. CIN can occur beyond 2 days, peaking on the fifth day. Therefore, some patients developing CIN beyond 48 hours have been missed. Fifth, an obvious source of conflict was that there is no general agreement on the safe dosage of NAC. In the trials of this meta‐analysis, NAC dosage ranged from 600–7200 mg/days; therefore, it is difficult to determine the optimal dose that would lead to the greatest improvement in renal function with limited side effects. Sixth, there is publication bias between studies, which questions the reliability of the results.

In conclusion, this meta‐analysis provides strong evidence that NAC supplementation is associated with a significantly lower risk of CIN. In real‐world practice, it is impossible to provide NAC for all patients undergoing contrast agent injection, while it is reasonable to administer NAC by the oral route for patients who are undergoing coronary angiography and who have renal dysfunction or who are receiving high doses of contrast agent. Additional randomized controlled trials with longer terms and larger populations are required to establish causality and to elucidate the underlying mechanisms.

## Author Contributions

R.‐F.X. and G.‐Z.C. conceived the study design, and wrote the manuscript; A.‐Y.T., Y.B., and Y.‐B.D. performed the analyses. All authors read and approved the final manuscript.

## Sources of Funding

The present study was supported by the National Natural Science Foundation of China (Nos. 81400369 and 81500293).

## Disclosures

None.

## Supporting information


**Table S1.** Quality assessment of included studies.
**Figure S1.** Meta‐analysis of effects for NAC (*N*‐acetylcysteine) on serum creatinine compared with control arms. IV indicates intravenous.
**Figure S2.** A, The association between NAC (*N*‐acetylcysteine) admission and the incidence of renal failure requiring renal dialysis. B, The association between NAC admission and the incidence of mortality. RR indicates risk ratio.
**Figure S3.** Funnel plot of *N*‐acetylcysteine consumption and contrast‐induced nephropathy. The SE of the risk ratio (RR) was plotted against the RR for contrast‐induced nephropathy.

## Notes

1 References 12, 29, 30, 31, 32, 33, 34, 35, 37, 38, 39, 40, 41, 42, 43, 46, 47, 48, 49, 50, 51, 53, 54, 55, 56, 57, 58, 59, 61, 63, 65, 66, 67, 68, 69, 71, 73, 76, 77, 78, 79, 80, 82, 83, 84, 86.

2 References 12, 29, 31, 33, 38, 39, 40, 44, 45, 46, 50, 51, 52, 56, 57, 58, 61, 62, 64, 66, 68, 69, 71, 72, 76, 81, 82, 83, 85, 86.

3 References 11, 30, 35, 37, 41, 43, 47, 48, 49, 59, 73, 75, 77.

4 References 32, 34, 36, 42, 53, 54, 55, 60, 63, 65, 67, 70, 74, 78, 79, 80, 84, 87.

5 References 32, 33, 34, 42, 44, 47, 50, 51, 53, 54, 59, 65, 66, 67, 69, 71, 77, 78, 79, 80, 81.

6 References 11, 12, 29, 30, 31, 35, 36, 37, 38, 39, 40, 41, 43, 45, 46, 48, 49, 52, 55, 56, 57, 58, 60, 61, 62, 63, 64, 68, 70, 72, 73, 74, 75, 76, 82, 83, 84, 85, 86, 87.

7 References 11, 12, 29, 30, 31, 33, 35, 36, 40, 41, 43, 44, 45, 46, 47, 48, 49, 50, 51, 53, 55, 57, 58, 59, 60, 61, 63, 64, 65, 66, 67, 68, 69, 73, 74, 75, 76, 77, 78, 79, 80, 83, 84, 85, 86, 87.

8 References 34, 37, 38, 39, 42, 44, 52, 54, 56, 62, 70, 72, 81, 82.

9 References 11, 12, 29, 30, 31, 33, 34, 35, 36, 37, 39, 40, 43, 44, 45, 46, 47, 48, 49, 50, 52, 53, 55, 58, 60, 61, 62, 64, 65, 67, 68, 69, 70, 72, 73, 75, 76, 77, 82, 86, 87.

10 References 32, 38, 41, 42, 51, 54, 56, 57, 59, 63, 66, 71, 74, 78, 79, 80, 81, 83, 84, 85.

11 References 12, 32, 34, 36, 38, 39, 40, 42, 45, 54, 56, 57, 58, 64, 66, 67, 73, 78, 80, 86.

12 References 11, 29, 30, 31, 35, 37, 41, 43, 44, 46, 47, 48, 50, 52, 53, 55, 59, 60, 61, 62, 65, 68, 69, 70, 72, 74, 75, 76, 77, 79, 81, 82, 83, 84, 85, 87.

13 References 11, 12, 31, 32, 33, 37, 38, 39, 40, 43, 45, 47, 48, 53, 54, 55, 56, 58, 60, 62, 64, 66, 68, 69, 70, 72, 76, 80, 81, 82, 86.

14 References 29, 30, 34, 35, 36, 41, 42, 44, 46, 49, 50, 51, 52, 57, 59, 61, 63, 65, 67, 71, 73, 74, 75, 77, 78, 79, 83, 84, 85, 87.

15 References 29, 31, 32, 33, 34, 35, 37, 39, 40, 42, 44, 45, 46, 48, 49, 50, 51, 52, 53, 54, 55, 56, 57, 59, 61, 62, 63, 64, 66, 68, 72, 74, 76, 79, 80, 81, 83, 84, 85, 87.

16 References 11, 12, 30, 36, 38, 41, 43, 47, 58, 65, 67, 69, 70, 77, 86.

17 References 31, 37, 38, 41, 43, 45, 46, 48, 53, 61, 67, 69, 72, 86.

18 References 11, 12, 29, 30, 31, 32, 33, 34, 35, 36, 37, 38, 39, 40, 41, 44, 45, 47, 48, 49, 50, 51, 52, 54, 55, 56, 57, 59, 60, 61, 63, 64, 65, 66, 67, 68, 70, 71, 73, 76, 77, 79, 80, 81, 83, 84, 85, 86, 87.

19 References 11, 34, 35, 36, 37, 38, 39, 40, 44, 46, 48, 49, 53, 55, 56, 57, 60, 64, 66, 67, 69, 71, 73, 77, 78, 79, 80, 82, 85, 86.

20 References 11, 34, 35, 37, 38, 39, 40, 41, 44, 46, 48, 53, 55, 56, 62, 64, 65, 66, 67, 68, 69, 70, 71, 73, 78, 80, 82.
