# Supplementary material for: Effectiveness of N‐Acetylcysteine for the Prevention of Contrast‐Induced Nephropathy: A Systematic Review and Meta‐Analysis of Randomized Controlled Trials
Source: J Am Heart Assoc. 2016 Sep 23;5(9):e003968. doi: 10.1161/JAHA.116.003968 (PMC5079043; doi:10.1161/JAHA.116.003968)
Supplement: Supplementary file 1 — Table S1. Quality assessment of included studies. Figure S1. Meta‐analysis of effects for NAC (N‐acetylcysteine) on serum creatinine compared with control arms. IV indicates intravenous. Figure S2. A, The association between NAC (N‐acetylcysteine) admission and the incidence of renal failure requiring renal dialysis. B, The association between NAC admission and the incidence of mortality. RR indicates risk ratio. Figure S3. Funnel plot of N‐acetylcysteine consumption and contrast‐induced nephropathy. The SE of the risk ratio (RR) was plotted against the RR for contrast‐induced nephropathy. [file JAH3-5-e003968-s001.pdf]

SUPPLEMENTAL MATERIAL

Table S1. Quality assessment of included studies.

First Author	Randomisation	Random sequence generation	Double blinding	Used identical placebo	Follow-up reporting	Total score
ACT et.al[1]	1	1	1	1	1	5
Albabtain et.al[2]	1	1	0	0	1	3
Allaqaband et.al[3]	1	1	0	0	1	3
Amini et.al[4]	1	1	1	1	1	5
Aslanger et.al[5]	1	1	0	1	1	4
Azmus et.al[6]	1	0	1	1	1	4
Baker et.al[7]	1	0	1	0	1	3
Baskurt et.al[8]	1	1	0	0	1	3
Briguori et.al[9]	1	0	0	0	1	2
Brueck et.al[10]	1	1	1	1	1	5
Carbonellet.al, 2007[11]	1	1	1	1	1	5
Carbonellet.al ,2010[12]	1	1	1	1	1	5
Castini et.al[13]	1	1	1	0	1	4
Coyle et.al[14]	1	1	0	0	1	3

Demir et.al[15]	1	0	0	0	1	2
Diaz-Sandoval et.al[16]	1	1	1	1	1	5
Droppa et.al[17]	1	0	0	1	1	3
Durham et.al[18]	1	1	0	1	1	4
Erturk et.al[19]	1	0	0	0	1	2
Ferrario et.al[20]	1	1	0	1	1	4
Fung et.al[21]	1	0	0	0	1	2
Goldenberg et.al[22]	1	1	1	1	1	5
Gomes et.al[23]	1	1	1	1	1	5
Gulel et.al[24]	1	1	0	0	1	3
Gunebakmaz et.al[25]	1	0	0	0	1	2
Habib et.al[26]	1	0	0	1	1	3
Hsu et.al ,2007[27]	1	1	0	1	1	4
Hsu et.al ,2012[28]	1	1	0	0	1	3
Jaffery et.al[29]	1	0	1	1	1	4
Kay et.al[30]	1	0	1	1	1	4
Kefer et.al[31]	1	0	1	1	1	4

Khalili et.al[32]	1	0	0	0	1	2
Kim et.al[33]	1	1	0	0	1	3
Kimmel et.al[34]	1	0	1	1	1	4
Kinbara et.al[35]	1	0	0	0	1	2
Kitzler et.al[36]	1	1	1	1	1	5
Koc et.al[37]	1	0	0	0	1	2
Kotlyar et.al[38]	1	1	1	1	1	5
Kumar et.al[39]	1	0	0	0	1	2
Lawlor et.al[40]	1	1	0	1	1	4
MacNeill et.al[41]	1	0	1	0	1	3
Marenzi et.al[42]	1	1	1	1	1	5
Miner et.al[43]	1	0	1	0	1	3
Ochoa et.al[44]	1	0	1	1	1	4
Oldemeyer et.al[45]	1	1	1	1	1	5
Poletti et.al[46]	1	0	1	1	1	4
Prasad et.al[47]	1	1	0	0	1	3
Rashid et.al[48]	1	0	1	1	1	4

Reinecke et.al[49]	1	0	0	0	1	2
Sadat et.al[50]	1	0	0	0	1	2
Sandhu et.al[51]	1	1	0	0	1	3
Seyon et.al[52]	1	0	1	1	1	4
Shyu et.al[53]	1	0	0	1	1	3
Tanaka et.al[54]	1	0	0	1	1	3
Tepel et.al[55]	1	0	1	1	1	4
Thayssen et.al[56]	1	1	0	0	1	3
Thiele et.al[57]	1	1	0	1	1	4
Traub et.al[58]	1	0	1	1	1	4
Webb et.al[59]	1	0	1	1	1	4
Yang et.al[60]	1	1	0	0	1	3
Yeganehkhah et.al[61]	1	1	0	0	1	3

Study or Subgroup	NAC Mean	NAC SD	NAC Total	Control Mean	Control SD	Control Total	Weight	Mean Difference IV, Random, 95% CI
Albabtain 2013	0.03	0.25	62	0.02	0.38	66	2.2%	0.01 [-0.10, 0.12]
Allaqaband 2002	0.01	0.62	45	0.09	0.3	40	1.6%	-0.08 [-0.28, 0.12]
Amini 2009	-0.02	0.36	45	-0.02	0.47	45	1.8%	0.00 [-0.17, 0.17]
Aslanger 2012	-0.016	0.363	45	-0.018	0.467	45	1.8%	0.00 [-0.17, 0.17]
Azmus 2005	1.3	0.43	196	1.38	0.39	201	2.4%	-0.08 [-0.16, 0.00]
Baker 2003	-0.08	0.32	41	0.09	0.29	39	2.1%	-0.17 [-0.30, -0.04]
Baskurt 2009	0.08	0.43	73	0.08	0.38	72	2.1%	0.00 [-0.13, 0.13]
Briguori 2002	-0.04	0.53	92	-0.01	0.58	91	1.9%	-0.03 [-0.19, 0.13]
Brueck 2013	0.15	0.31	199	0.2	0.35	198	2.5%	-0.05 [-0.12, 0.02]
Carbonell 2007	0.05	0.26	107	0.03	0.26	109	2.5%	0.02 [-0.05, 0.09]
Carbonell 2009	-0.11	1.11	39	0.28	1.12	42	0.6%	-0.39 [-0.88, 0.10]
Castini 2008	-0.08	0.22	53	0.12	0.24	51	2.4%	-0.20 [-0.29, -0.11]
Coyle 2006	0.15	0.42	65	0.08	0.11	69	2.3%	0.07 [-0.04, 0.18]
Demir 2008	0.02	0.23	20	-0.01	0.11	20	2.2%	0.03 [-0.08, 0.14]
Diaz-Sandoval 2001	-0.1	0.06	25	0.3	0.06	29	2.6%	-0.40 [-0.43, -0.37]
Erturk 2014	0.04	0.61	102	0.06	0.77	103	1.7%	-0.02 [-0.21, 0.17]
Erturk-b 2014	0.01	0.56	102	0.06	0.77	103	1.8%	-0.05 [-0.23, 0.13]
Fung 2004	0.17	0.39	46	0.07	0.4	45	1.9%	0.10 [-0.06, 0.26]
Goldenberg 2003	0.01	0.36	41	0.03	0.32	39	2.0%	-0.02 [-0.17, 0.13]
Gomes 2005	0.09	0.4	77	0.07	0.29	79	2.3%	0.02 [-0.09, 0.13]
Gulel 2005	-0.098	0.14	25	-0.017	0.14	25	2.4%	-0.08 [-0.16, -0.00]
Gunebakmaz 2012	0.1	0.26	40	0.12	0.28	40	2.2%	-0.02 [-0.14, 0.10]
Habib 2016	-0.02	0.62	30	0.13	0.43	45	1.3%	-0.15 [-0.40, 0.10]
Hsu 2007	-0.4	1.15	11	-0.2	0.86	9	0.2%	-0.20 [-1.08, 0.68]
Hsu 2012	0.05	0.93	106	0.32	1.02	103	1.3%	-0.27 [-0.53, -0.01]
Jaffery 2011	0.07	0.58	206	0.06	0.56	192	2.2%	0.01 [-0.10, 0.12]
Kay 2003	-0.13	0.47	98	0.02	0.13	102	2.3%	-0.15 [-0.25, -0.05]
Kefer 2003	-0.01	0.36	53	-0.1	0.67	51	1.6%	0.09 [-0.12, 0.30]
Khalili 2006	0.02	0.02	35	0.23	0.07	35	2.6%	-0.21 [-0.23, -0.19]
Kimmel 2008	-0.03	0.31	19	-0.2	0.64	17	1.0%	0.17 [-0.16, 0.50]
Kinbara 2009	-0.33	0.42	15	0.34	0.31	15	1.3%	-0.67 [-0.93, -0.41]
Kitzler 2012	0.02	0.11	10	-0.02	0.13	10	2.3%	0.04 [-0.07, 0.15]
Koc 2010	-0.04	0.15	80	0.11	0.34	60	2.4%	-0.15 [-0.24, -0.06]
Kumar 2014	0.23	0.07	40	0.3	0.07	40	2.6%	-0.07 [-0.10, -0.04]
Kumar-b 2014	0.31	0.08	50	0.38	0.1	50	2.6%	-0.07 [-0.11, -0.03]
Lawlor 2007	0.07	0.85	25	0.09	0.82	25	0.6%	-0.02 [-0.48, 0.44]
Lawlor-b 2007	0.07	0.8	28	0.09	0.82	25	0.7%	-0.02 [-0.46, 0.42]
MacNeill 2003	0.01	0.48	21	0.5	1.2	22	0.5%	-0.49 [-1.03, 0.05]
Marenzi 2006	-0.05	0.16	115	0.04	0.22	119	2.6%	-0.09 [-0.14, -0.04]
Marenzi-b 2006	-0.05	0.16	118	0.04	0.22	119	2.6%	-0.09 [-0.14, -0.04]
Miner 2004	-0.06	0.29	95	0.12	0.16	85	2.5%	-0.18 [-0.25, -0.11]
Ochoa 2004	0.08	0.5	36	0.17	0.42	44	1.6%	-0.09 [-0.30, 0.12]
Poletti 2007	-0.18	0.36	43	-0.01	0.46	44	1.8%	-0.17 [-0.34, 0.00]
Prasad 2010	-0.03	0.26	35	-0.06	0.32	35	2.1%	0.03 [-0.11, 0.17]
Reinecke 2006	0.24	0.43	140	0.14	0.24	146	2.4%	0.10 [0.02, 0.18]
Seyon 2006	0.11	0.68	20	0.11	0.62	20	0.8%	0.00 [-0.40, 0.40]
Shyu 2002	-0.29	0.41	60	0.24	0.56	61	1.8%	-0.53 [-0.70, -0.36]
Tepel 2000	-0.4	0.8	41	0.2	0.9	42	0.9%	-0.60 [-0.97, -0.23]
Thayseen 2014	0.06	0.24	176	0.07	0.28	181	2.6%	-0.01 [-0.06, 0.04]
Thiele 2010	-1.84	15.71	126	2	19.88	125	0.0%	-3.84 [-8.28, 0.60]
Traub 2013	-0.05	0.25	185	-0.025	0.23	172	2.6%	-0.03 [-0.07, 0.02]
Yang 2015	0.04	0.32	157	0.04	0.32	161	2.5%	0.00 [-0.07, 0.07]
Yeganehkhah 2014	-0.06	0.31	50	0.05	0.17	50	2.3%	-0.11 [-0.21, -0.01]
**Total (95% CI)**			**3764**			**3756**	**100.0%**	**-0.08 [-0.12, -0.04]**

Heterogeneity: Tau² = 0.02; Chi² = 594.99, df = 52 (P < 0.00001); I² = 91%

Test for overall effect: Z = 3.93 (P < 0.0001)

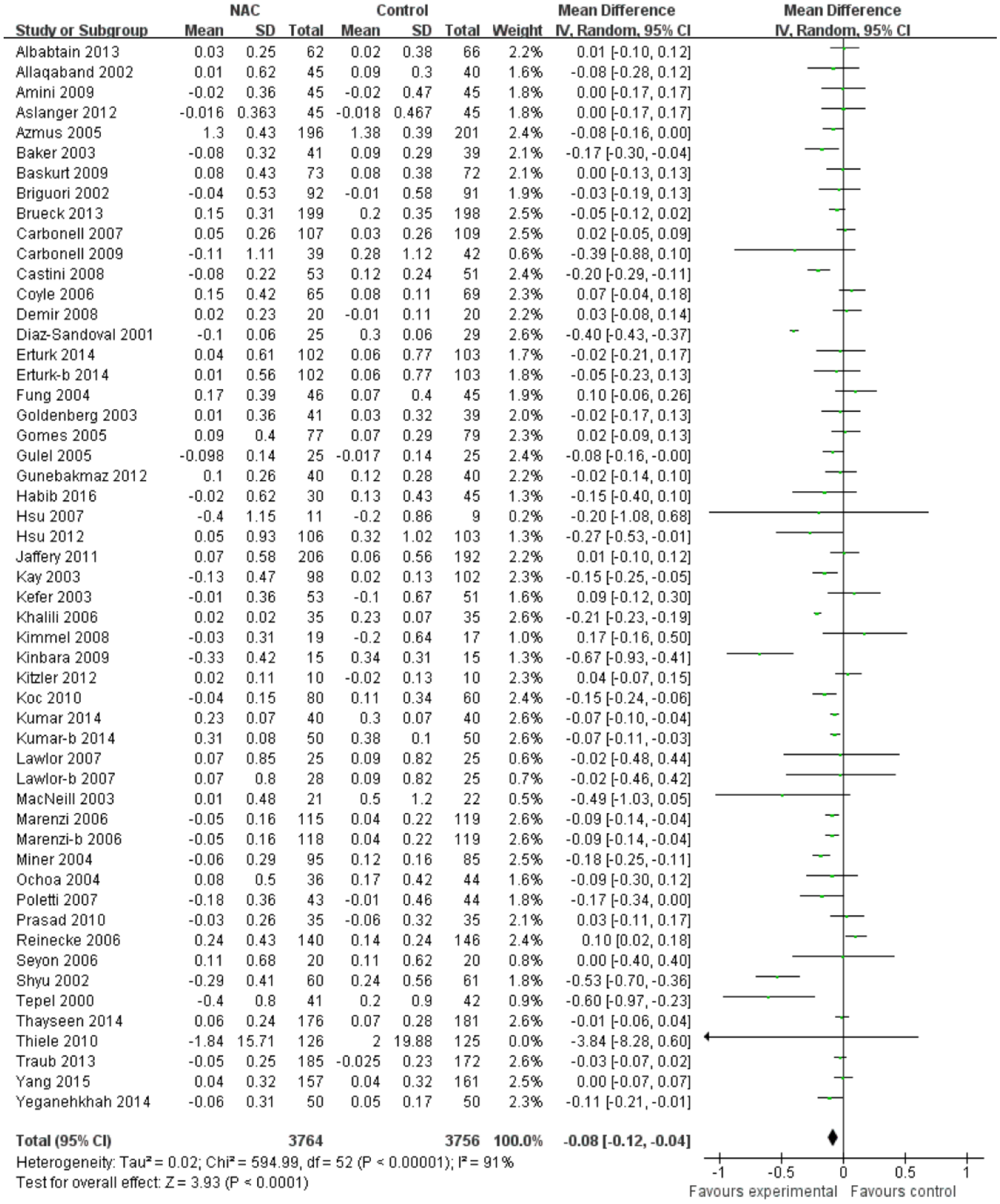

Figure S1. Meta-analysis of effects for NAC (N-acetylcysteine) on serum creatitine compared with control arms. IV: intravenous; CI, confidence interval.

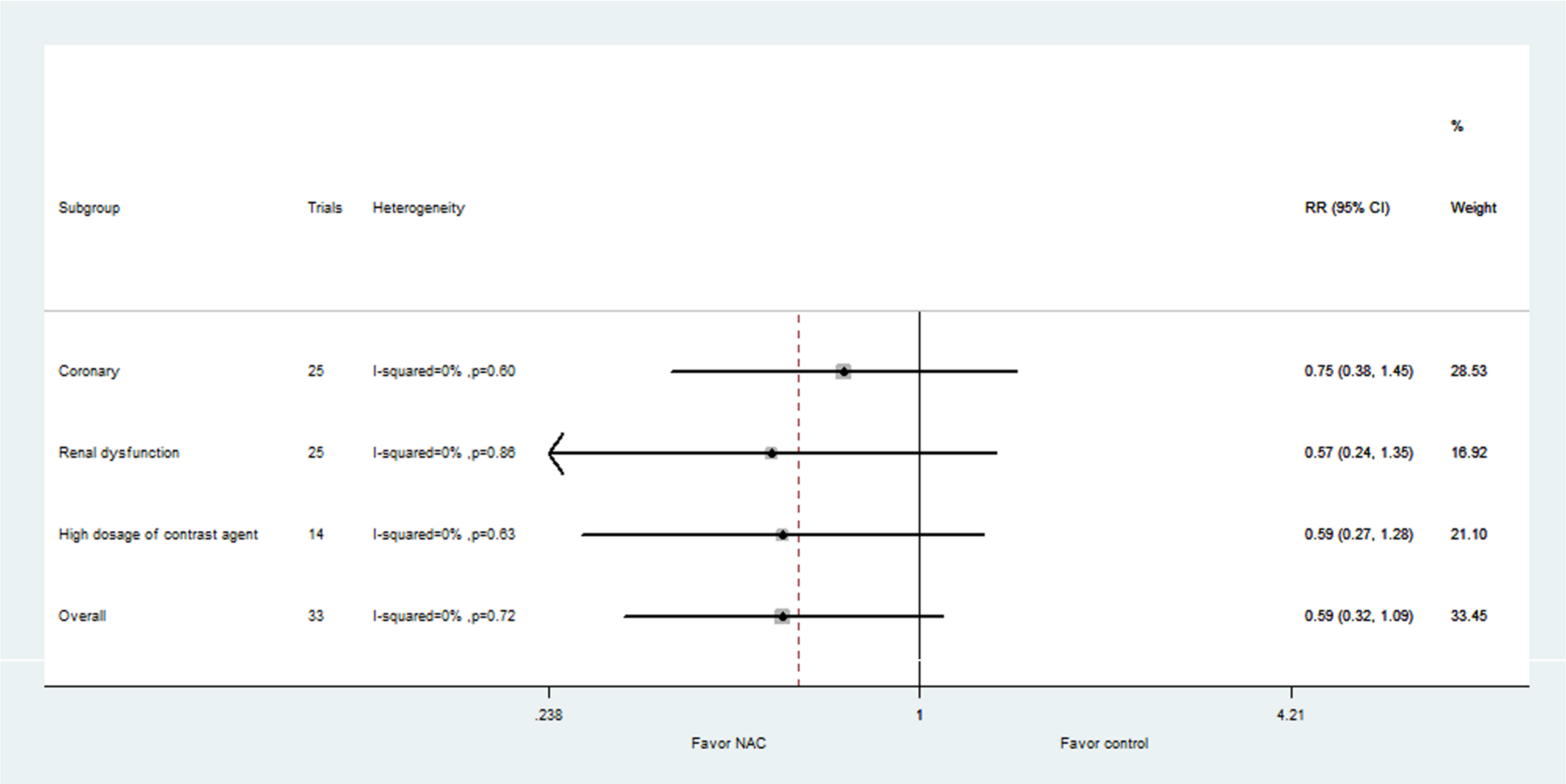
%
Subgroup
Trials
Heterogeneity
RR (95% CI)
Weight
Coronary
25
I-squared=0% ,p=0.60
0.75 (0.38, 1.45)
28.53
Renal dysfunction
25
I-squared=0% ,p=0.86
0.57 (0.24, 1.35)
16.92
High dosage of contrast agent
14
I-squared=0% ,p=0.63
0.59 (0.27, 1.28)
21.10
Overall
33
I-squared=0% ,p=0.72
0.59 (0.32, 1.09)
33.45
.238
1
4.21
Favor NAC
Favor control

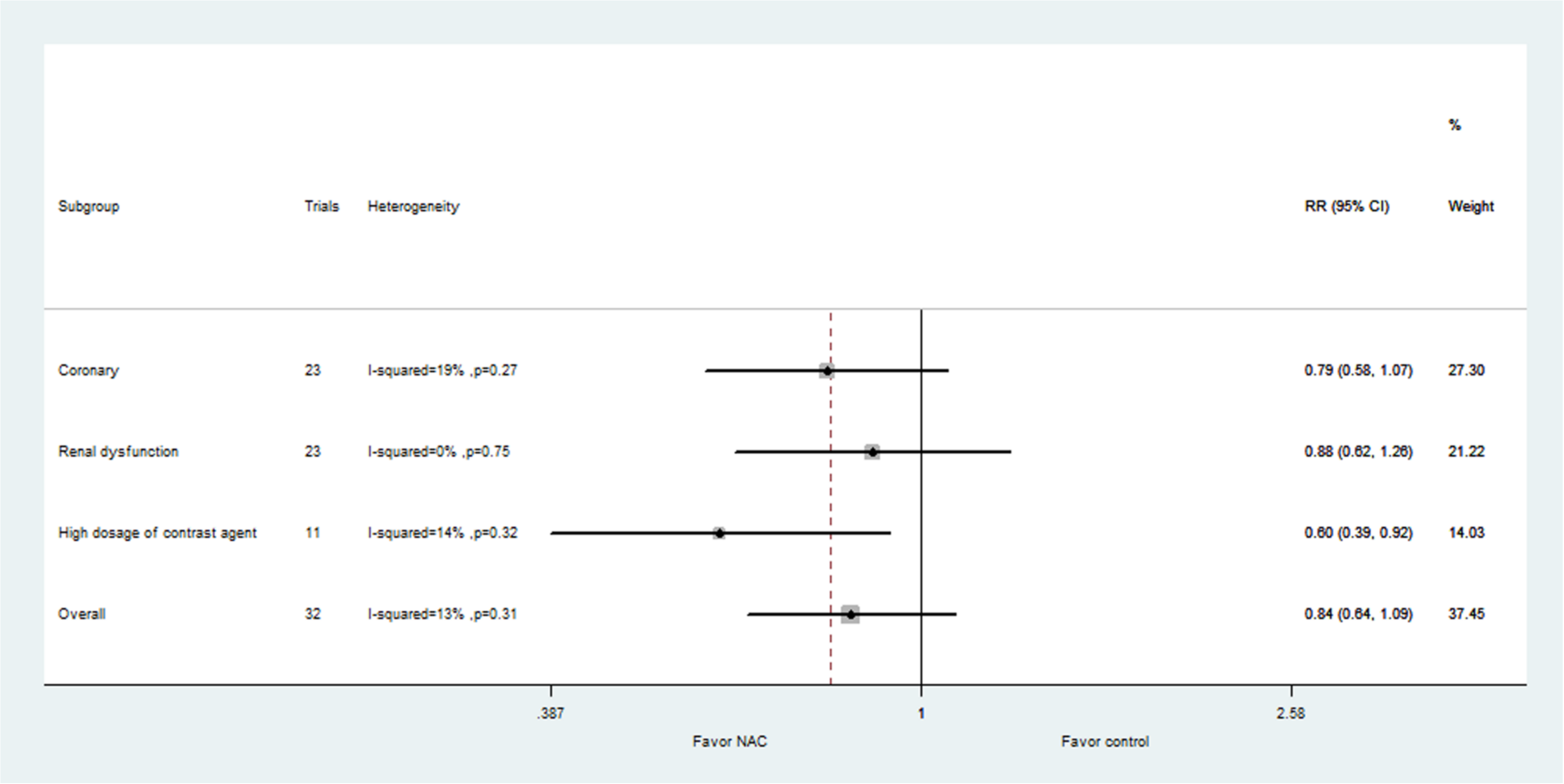

Figure S2. A: The association between NAC admission and the incidence of renal failure requiring renal dialysis. B: The association between NAC admission and the incidence of mortality. RR, risk ratio; CI, confidence interval.

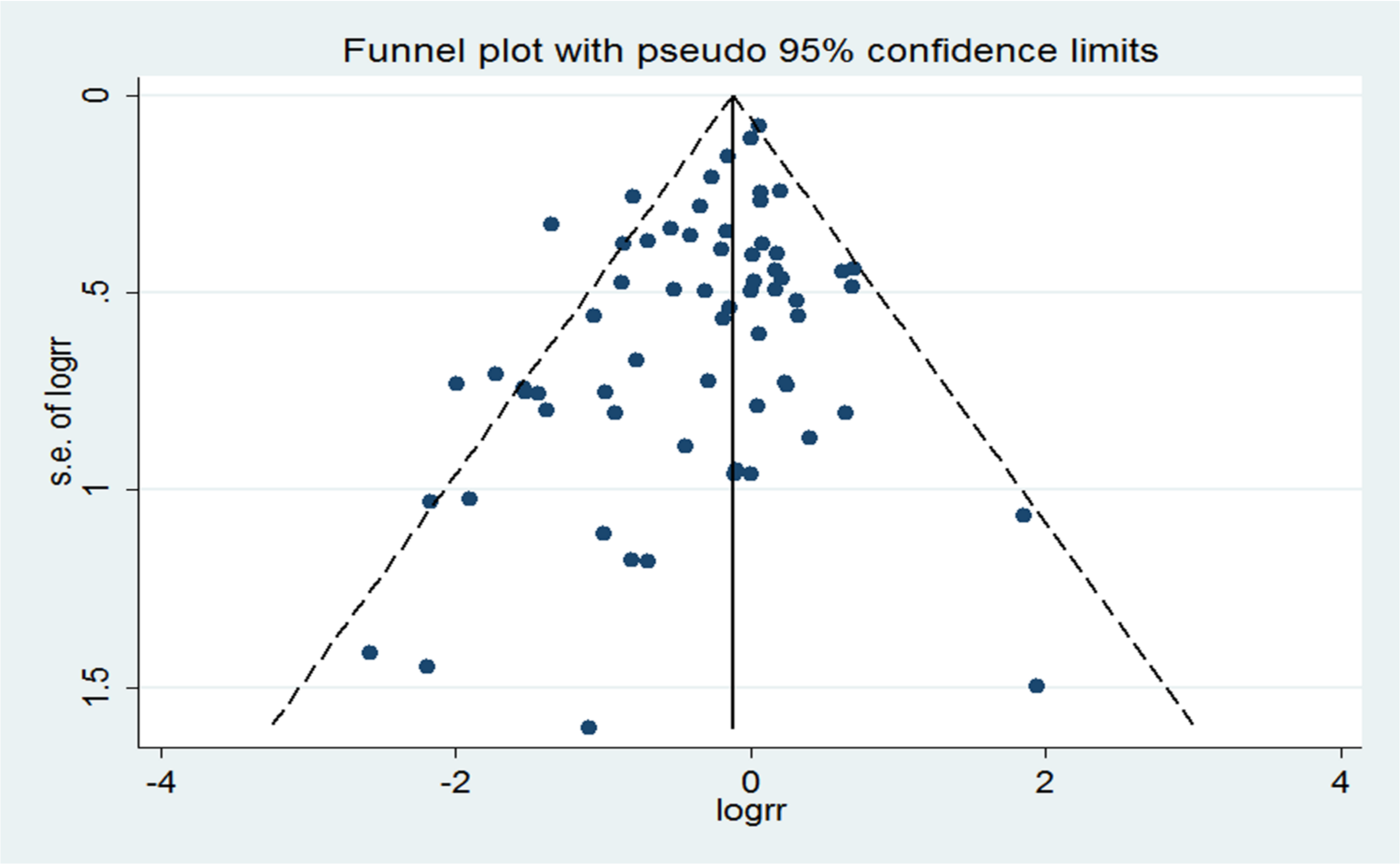

Figure S3. Funnel Plot of N-acetylcysteine Consumption and contrast-induced nephropathy. The standard error (SE) of the Risk ratio (RR) was plotted against the RR for contrast-induced nephropathy.

References

1. Investigators ACT. Acetylcysteine for prevention of renal outcomes in patients undergoing coronary and peripheral vascular angiography: Main results from the randomized acetylcysteine for contrast-induced nephropathy trial (act). *Circulation*. 2011;124:1250-1259
2. Albabtain MA, Almasood A, Alshurafah H, Alamri H, Tamim H. Efficacy of ascorbic acid, n-acetylcysteine, or combination of both on top of saline hydration versus saline hydration alone on prevention of contrast-induced nephropathy: A prospective randomized study. *Journal of interventional cardiology*. 2013;26:90-96
3. Allaqaband S, Tumuluri R, Malik AM, Gupta A, Volkert P, Shalev Y, Bajwa TK. Prospective randomized study of n-acetylcysteine, fenoldopam, and saline for prevention of radiocontrast-induced nephropathy. *Catheterization and cardiovascular interventions : official journal of the Society for Cardiac Angiography & Interventions*. 2002;57:279-283
4. Amini M, Salarifar M, Amirbaigloo A, Masoudkabir F, Esfahani F. N-acetylcysteine does not prevent contrast-induced nephropathy after cardiac catheterization in patients with diabetes mellitus and chronic kidney disease: A randomized clinical trial. *Trials*. 2009;10:45
5. Aslanger E, Uslu B, Akdeniz C, Polat N, Cizgici Y, Oflaz H. Intrarenal application of n-acetylcysteine for the prevention of contrast medium-induced nephropathy in primary angioplasty. *Coronary artery disease*. 2012;23:265-270
6. Azmus AD, Gottschall C, Manica A, Manica J, Duro K, Frey M, Bulcao L, Lima C. Effectiveness of acetylcysteine in prevention of contrast nephropathy. *The Journal of invasive cardiology*. 2005;17:80-84
7. Baker CS, Wragg A, Kumar S, De Palma R, Baker LR, Knight CJ. A rapid protocol for the prevention of contrast-induced renal dysfunction: The rappid study. *Journal of the American College of Cardiology*. 2003;41:2114-2118
8. Baskurt M, Okcun B, Abaci O, Dogan GM, Kilickesmez K, Ozkan AA, Ersanli M, Gurmen T. N-acetylcysteine versus n-acetylcysteine + theophylline for the prevention of contrast nephropathy. *European journal of clinical investigation*. 2009;39:793-799
9. Briguori C, Manganelli F, Scarpato P, Elia PP, Golia B, Riviezzo G, Lepore S, Librera M, Villari B, Colombo A, Ricciardelli B. Acetylcysteine and contrast agent-associated nephrotoxicity. *Journal of the American College of Cardiology*. 2002;40:298-303
10. Brueck M, Cengiz H, Hoeltgen R, Wieczorek M, Boedeker RH, Scheibelhut C, Boening A. Usefulness of n-acetylcysteine or ascorbic acid versus placebo to prevent contrast-induced acute kidney injury in patients undergoing elective cardiac catheterization: A single-center, prospective, randomized, double-blind, placebo-controlled trial. *The Journal of invasive cardiology*. 2013;25:276-283
11. Carbonell N, Blasco M, Sanjuan R, Perez-Sancho E, Sanchis J, Insa L, Bodi V, Nunez J, Garcia-Ramon R, Miguel A. Intravenous n-acetylcysteine for preventing contrast-induced nephropathy: A randomised trial. *International journal of cardiology*. 2007;115:57-62
12. Carbonell N, Sanjuan R, Blasco M, Jorda A, Miguel A. N-acetylcysteine: Short-term clinical benefits after coronary angiography in high-risk renal patients. *Revista espanola de cardiologia*. 2010;63:12-19

13. Castini D, Lucreziotti S, Bosotti L, Salerno Uriarte D, Sponzilli C, Verzoni A, Lombardi F. Prevention of contrast-induced nephropathy: A single center randomized study. *Clinical cardiology*. 2010;33:E63-68
14. Coyle LC, Rodriguez A, Jeschke RE, Simon-Lee A, Abbott KC, Taylor AJ. Acetylcysteine in diabetes (aid): A randomized study of acetylcysteine for the prevention of contrast nephropathy in diabetics. *American heart journal*. 2006;151:1032 e1039-1012
15. Demir M, Kutlucan A, Akin H, Aydin O, Sezer T. Comparison of different agents on radiographic contrast agent induced nephropathy .Eur J Gen Med 2008;5:222-227
16. Diaz-Sandoval LJ, Kosowsky BD, Losordo DW. Acetylcysteine to prevent angiography-related renal tissue injury (the apart trial). *The American journal of cardiology*. 2002;89:356-358
17. Droppa M, Desch S, Blase P, Eitel I, Fuernau G, Schuler G, Adams V, Thiele H. Impact of n-acetylcysteine on contrast-induced nephropathy defined by cystatin c in patients with st-elevation myocardial infarction undergoing primary angioplasty. *Clinical research in cardiology : official journal of the German Cardiac Society*. 2011;100:1037-1043
18. Durham JD, Caputo C, Dokko J, Zaharakis T, Pahlavan M, Keltz J, Dutka P, Marzo K, Maesaka JK, Fishbane S. A randomized controlled trial of n-acetylcysteine to prevent contrast nephropathy in cardiac angiography. *Kidney international*. 2002;62:2202-2207
19. Erturk M, Uslu N, Gorgulu S, Akbay E, Kurtulus G, Akturk IF, Akgul O, Surgit O, Uzun F, Gul M, Isiksacan N, Yildirim A. Does intravenous or oral high-dose n-acetylcysteine in addition to saline prevent contrast-induced nephropathy assessed by cystatin c? *Coronary artery disease*. 2014;25:111-117
20. Ferrario F, Barone MT, Landoni G, Genderini A, Heidemperger M, Trezzi M, Piccaluga E, Danna P, Scorza D. Acetylcysteine and non-ionic isosmolar contrast-induced nephropathy--a randomized controlled study. *Nephrology, dialysis, transplantation : official publication of the European Dialysis and Transplant Association - European Renal Association*. 2009;24:3103-3107
21. Fung JW, Szeto CC, Chan WW, Kum LC, Chan AK, Wong JT, Wu EB, Yip GW, Chan JY, Yu CM, Woo KS, Sanderson JE. Effect of n-acetylcysteine for prevention of contrast nephropathy in patients with moderate to severe renal insufficiency: A randomized trial. *American journal of kidney diseases : the official journal of the National Kidney Foundation*. 2004;43:801-808
22. Goldenberg I, Shechter M, Matetzky S, Jonas M, Adam M, Pres H, Elian D, Agranat O, Schwammenthal E, Guetta V. Oral acetylcysteine as an adjunct to saline hydration for the prevention of contrast-induced nephropathy following coronary angiography. A randomized controlled trial and review of the current literature. *European heart journal*. 2004;25:212-218
23. Gomes VO, Poli de Figueredo CE, Caramori P, Lasevitch R, Bodanese LC, Araujo A, Roedel AP, Caramori AP, Brito FS, Jr., Bezerra HG, Nery P, Brizolara A. N-acetylcysteine does not prevent contrast induced nephropathy after cardiac catheterisation with an ionic low osmolality contrast medium: A multicentre clinical trial. *Heart*. 2005;91:774-778
24. Gulel O, Keles T, Eraslan H, Aydogdu S, Diker E, Ulusoy V. Prophylactic acetylcysteine usage for prevention of contrast nephropathy after coronary angiography. *Journal of cardiovascular pharmacology*. 2005;46:464-467
25. Gunebakmaz O, Kaya MG, Koc F, Akpek M, Kasapkara A, Inanc MT, Yarlioglues M, Calapkorur B, Karadag Z, Oguzhan A. Does nebivolol prevent

contrast-induced nephropathy in humans? *Clinical cardiology*. 2012;35:250-254
26. Habib M, Hillis A, Hammad A. N-acetylcysteine and/or ascorbic acid versus placebo to prevent contrast-induced nephropathy in patients undergoing elective cardiac catheterization: The napcin trial; a single-center, prospective, randomized trial. *Saudi journal of kidney diseases and transplantation : an official publication of the Saudi Center for Organ Transplantation, Saudi Arabia*. 2016;27:55-61
27. Hsu CH, Lee JD, Lo PH, Lin JJ, Chang HW, Chou HT. Prevention of radiocontrast-induced nephropathy with n-acetylcysteine after cardiac angiography in diabetic patients with renal dysfunction. *Mid Taiwan J Med 2007;12:173-83*
28. Hsu TF, Huang MK, Yu SH, Yen DH, Kao WF, Chen YC, Huang MS. N-acetylcysteine for the prevention of contrast-induced nephropathy in the emergency department. *Internal medicine*. 2012;51:2709-2714
29. Jaffery Z, Verma A, White CJ, Grant AG, Collins TJ, Grise MA, Jenkins JS, McMullan PW, Patel RA, Reilly JP, Thornton SN, Ramee SR. A randomized trial of intravenous n-acetylcysteine to prevent contrast induced nephropathy in acute coronary syndromes. *Catheterization and cardiovascular interventions : official journal of the Society for Cardiac Angiography & Interventions*. 2012;79:921-926
30. Kay J, Chow WH, Chan TM, Lo SK, Kwok OH, Yip A, Fan K, Lee CH, Lam WF. Acetylcysteine for prevention of acute deterioration of renal function following elective coronary angiography and intervention: A randomized controlled trial. *Jama*. 2003;289:553-558
31. Kefer JM, Hanet CE, Boitte S, Wilmotte L, De Kock M. Acetylcysteine, coronary procedure and prevention of contrast-induced worsening of renal function: Which benefit for which patient? *Acta cardiologica*. 2003;58:555-560
32. Khalili H, Dashti-Khavidaki S. Tabifar H, Ahmadinejad N,Ahmadi F. N-acetylcysteine in the prevention of contrast agent -induced nephrotoxicity in patients undergoing computed tomography studies. Therapy 2006;3:773-777
33. Kim BJ, Sung KC, Kim BS, Kang JH, Lee KB, Kim H, Lee MH. Effect of n-acetylcysteine on cystatin c-based renal function after elective coronary angiography (enable study): A prospective, randomized trial. *International journal of cardiology*. 2010;138:239-245
34. Kimmel M, Butscheid M, Brenner S, Kuhlmann U, Klotz U, Alscher DM. Improved estimation of glomerular filtration rate by serum cystatin c in preventing contrast induced nephropathy by n-acetylcysteine or zinc--preliminary results. *Nephrology, dialysis, transplantation : official publication of the European Dialysis and Transplant Association - European Renal Association*. 2008;23:1241-1245
35. Kinbara T, Hayano T, Ohtani N, Furutani Y, Moritani K, Matsuzaki M. Efficacy of n-acetylcysteine and aminophylline in preventing contrast-induced nephropathy. *Journal of cardiology*. 2010;55:174-179
36. Kitzler TM, Jaberi A, Sendlhofer G, Rehak P, Binder C, Petnehazy E, Stacher R, Kotanko P. Efficacy of vitamin e and n-acetylcysteine in the prevention of contrast induced kidney injury in patients with chronic kidney disease: A double blind, randomized controlled trial. *Wiener klinische Wochenschrift*. 2012;124:312-319
37. Koc F, Ozdemir K, Kaya MG, Dogdu O, Vatankulu MA, Ayhan S, Erkorkmaz U, Sonmez O, Aygul MU, Kalay N, Kayrak M, Karabag T, Alihanoglu Y,

Gunebakmaz O. Intravenous n-acetylcysteine plus high-dose hydration versus high-dose hydration and standard hydration for the prevention of contrast-induced nephropathy: Casis--a multicenter prospective controlled trial. *International journal of cardiology*. 2012;155:418-423
38. Kotlyar E, Keogh AM, Thavapalachandran S, Allada CS, Sharp J, Dias L, Muller D. Prehydration alone is sufficient to prevent contrast-induced nephropathy after day-only angiography procedures--a randomised controlled trial. *Heart, lung & circulation*. 2005;14:245-251
39. Kumar A, Bhawani G, Kumari N, Murthy KS, Lalwani V, Raju Ch N. Comparative study of renal protective effects of allopurinol and n-acetyl-cysteine on contrast induced nephropathy in patients undergoing cardiac catheterization. *Journal of clinical and diagnostic research : JCDR*. 2014;8:HC03-07
40. Lawlor DK, Moist L, DeRose G, Harris KA, Lovell MB, Kribs SW, Elliot J, Forbes TL. Prevention of contrast-induced nephropathy in vascular surgery patients. *Annals of vascular surgery*. 2007;21:593-597
41. MacNeill BD, Harding SA, Bazari H, Patton KK, Colon-Hernadez P, DeJoseph D, Jang IK. Prophylaxis of contrast-induced nephropathy in patients undergoing coronary angiography. *Catheterization and cardiovascular interventions : official journal of the Society for Cardiac Angiography & Interventions*. 2003;60:458-461
42. Marenzi G, Assanelli E, Marana I, Lauri G, Campodonico J, Grazi M, De Metrio M, Galli S, Fabbiocchi F, Montorsi P, Veglia F, Bartorelli AL. N-acetylcysteine and contrast-induced nephropathy in primary angioplasty. *The New England journal of medicine*. 2006;354:2773-2782
43. Miner SE, Dzavik V, Nguyen-Ho P, Richardson R, Mitchell J, Atchison D, Seidelin P, Daly P, Ross J, McLaughlin PR, Ing D, Lewycky P, Barolet A, Schwartz L. N-acetylcysteine reduces contrast-associated nephropathy but not clinical events during long-term follow-up. *American heart journal*. 2004;148:690-695
44. Ochoa A, Pellizzon G, Addala S, Grines C, Isayenko Y, Boura J, Rempinski D, O'Neill W, Kahn J. Abbreviated dosing of n-acetylcysteine prevents contrast-induced nephropathy after elective and urgent coronary angiography and intervention. *Journal of interventional cardiology*. 2004;17:159-165
45. Oldemeyer JB, Biddle WP, Wurdeman RL, Mooss AN, Cichowski E, Hilleman DE. Acetylcysteine in the prevention of contrast-induced nephropathy after coronary angiography. *American heart journal*. 2003;146:E23
46. Poletti PA, Saudan P, Platon A, Mermillod B, Sautter AM, Vermeulen B, Sarasin FP, Becker CD, Martin PY. I.V. N-acetylcysteine and emergency ct: Use of serum creatinine and cystatin c as markers of radiocontrast nephrotoxicity. *AJR. American journal of roentgenology*. 2007;189:687-692
47. Prasad A, Banakal S, Muralidhar K. N-acetylcysteine does not prevent renal dysfunction after off-pump coronary artery bypass surgery. *European journal of anaesthesiology*. 2010;27:973-977
48. Rashid ST, Salman M, Myint F, Baker DM, Agarwal S, Sweny P, Hamilton G. Prevention of contrast-induced nephropathy in vascular patients undergoing angiography: A randomized controlled trial of intravenous n-acetylcysteine. *Journal of vascular surgery*. 2004;40:1136-1141
49. Reinecke H, Fobker M, Wellmann J, Becke B, Fleiter J, Heitmeyer C, Breithardt G, Hense HW, Schaefer RM. A randomized controlled trial comparing hydration therapy to additional hemodialysis or n-acetylcysteine for the prevention of contrast medium-induced nephropathy: The dialysis-

versus-diuresis (dvd) trial. *Clinical research in cardiology : official journal of the German Cardiac Society*. 2007;96:130-139
50. Sadat U, Walsh SR, Norden AG, Gillard JH, Boyle JR. Does oral n-acetylcysteine reduce contrast-induced renal injury in patients with peripheral arterial disease undergoing peripheral angiography? A randomized-controlled study. *Angiology*. 2011;62:225-230
51. Sandhu C, Belli AM, Oliveira DB. The role of n-acetylcysteine in the prevention of contrast-induced nephrotoxicity. *Cardiovascular and interventional radiology*. 2006;29:344-347
52. Seyon RA, Jensen LA, Ferguson IA, Williams RG. Efficacy of n-acetylcysteine and hydration versus placebo and hydration in decreasing contrast-induced renal dysfunction in patients undergoing coronary angiography with or without concomitant percutaneous coronary intervention. *Heart & lung : the journal of critical care*. 2007;36:195-204
53. Shyu KG, Cheng JJ, Kuan P. Acetylcysteine protects against acute renal damage in patients with abnormal renal function undergoing a coronary procedure. *Journal of the American College of Cardiology*. 2002;40:1383-1388
54. Tanaka A, Suzuki Y, Suzuki N, Hirai T, Yasuda N, Miki K, Fujita M, Tanaka T. Does n-acetylcysteine reduce the incidence of contrast-induced nephropathy and clinical events in patients undergoing primary angioplasty for acute myocardial infarction? *Internal medicine*. 2011;50:673-677
55. Tepel M, van der Giet M, Schwarzfeld C, Laufer U, Liermann D, Zidek W. Prevention of radiographic-contrast-agent-induced reductions in renal function by acetylcysteine. *The New England journal of medicine*. 2000;343:180-184
56. Thayssen P, Lassen JF, Jensen SE, Hansen KN, Hansen HS, Christiansen EH, Junker A, Ravkilde J, Thuesen L, Veien KT, Jensen LO. Prevention of contrast-induced nephropathy with n-acetylcysteine or sodium bicarbonate in patients with st-segment-myocardial infarction: A prospective, randomized, open-labeled trial. *Circulation. Cardiovascular interventions*. 2014;7:216-224
57. Thiele H, Hildebrand L, Schirdewahn C, Eitel I, Adams V, Fuernau G, Erbs S, Linke A, Diederich KW, Nowak M, Desch S, Gutberlet M, Schuler G. Impact of high-dose n-acetylcysteine versus placebo on contrast-induced nephropathy and myocardial reperfusion injury in unselected patients with st-segment elevation myocardial infarction undergoing primary percutaneous coronary intervention. The lipsia-n-acc (prospective, single-blind, placebo-controlled, randomized leipzig immediate percutaneous coronary intervention acute myocardial infarction n-acc) trial. *Journal of the American College of Cardiology*. 2010;55:2201-2209
58. Traub SJ, Mitchell AM, Jones AE, Tang A, O'Connor J, Nelson T, Kellum J, Shapiro NI. N-acetylcysteine plus intravenous fluids versus intravenous fluids alone to prevent contrast-induced nephropathy in emergency computed tomography. *Annals of emergency medicine*. 2013;62:511-520 e525
59. Webb JG, Pate GE, Humphries KH, Buller CE, Shalansky S, Al Shamari A, Sutander A, Williams T, Fox RS, Levin A. A randomized controlled trial of intravenous n-acetylcysteine for the prevention of contrast-induced nephropathy after cardiac catheterization: Lack of effect. *American heart journal*. 2004;148:422-429
60. Yang K, Liu W, Ren W, Lv S. Different interventions in preventing contrast-induced nephropathy after percutaneous coronary intervention. *International urology and nephrology*. 2014;46:1801-1807

61. Yeganehkhah MR, Iranirad L, Dorri F, Pazoki S, Akbari H, Miryounesi M, Vahedian M, Nazeri A, Hosseinzadeh F, Vafaeimanesh J. Comparison between three supportive treatments for prevention of contrast-induced nephropathy in high-risk patients undergoing coronary angiography. *Saudi journal of kidney diseases and transplantation : an official publication of the Saudi Center for Organ Transplantation, Saudi Arabia*. 2014;25:1217-1223
